# Critical Review of Lipid-Based Nanoparticles as Carriers of Neuroprotective Drugs and Extracts

**DOI:** 10.3390/nano11030563

**Published:** 2021-02-24

**Authors:** Filipe Fernandes, Mónica Dias-Teixeira, Cristina Delerue-Matos, Clara Grosso

**Affiliations:** 1 REQUIMTE/LAQV, Instituto Superior de Engenharia do Instituto Politécnico do Porto, Rua Dr. António Bernardino de Almeida, 431, 4249-015 Porto, Portugal; filiperw@hotmail.com (F.F.); monica.teixeira@erisa.pt (M.D.-T.); cmm@isep.ipp.pt (C.D.-M.); 2 NICiTeS—Núcleo de Investigação em Ciências e Tecnologias da Saúde, Escola Superior de Saúde Ribeiro Sanches, 1649-028 Lisbon, Portugal

**Keywords:** blood-brain barrier, lipids, nanoparticles, nanoemulsions, nanoliposomes, nanophytosomes, natural products, solid lipid nanoparticles, nanostructured lipid carriers

## Abstract

The biggest obstacle to the treatment of diseases that affect the central nervous system (CNS) is the passage of drugs across the blood-brain barrier (BBB), a physical barrier that regulates the entry of substances into the brain and ensures the homeostasis of the CNS. This review summarizes current research on lipid-based nanoparticles for the nanoencapsulation of neuroprotective compounds. A survey of studies on nanoemulsions (NEs), nanoliposomes/nanophytosomes and solid lipid nanoparticles (SLNs)/nanostructured lipid carriers (NLCs) was carried out and is discussed herein, with particular emphasis upon their unique characteristics, the most important parameters influencing the formulation of each one, and examples of neuroprotective compounds/extracts nanoencapsulated using these nanoparticles. Gastrointestinal absorption is also discussed, as it may pose some obstacles for the absorption of free and nanoencapsulated neuroprotective compounds into the bloodstream, consequently hampering drug concentration in the brain. The transport mechanisms through which compounds or nanoparticles may cross BBB into the brain parenchyma, and the potential to increase drug bioavailability, are also discussed. Additionally, factors contributing to BBB disruption and neurodegeneration are described. Finally, the advantages of, and obstacles to, conventional and unconventional routes of administration to deliver nanoencapsulated neuroprotective drugs to the brain are also discussed, taking into account the avoidance of first-pass metabolism, onset of action, ability to bypass the BBB and concentration of the drug in the brain.

## 1. Introduction

Neurological disorders are the leading cause of disability and the second highest cause of death worldwide, with the numbers being expected to rise in the coming decades. This has led to an increase in both the number of deaths and the years lived with disability [1]. The main obstacle in treating these disorders is the administration of pharmaceuticals to the brain parenchyma. Three direct routes can be considered: delivery across the blood-brain barrier (BBB), intranasal delivery and intrathecal delivery; among them, delivery across the BBB is the most studied and appealing due to its minimally invasive nature [2].

Encapsulation can be defined “as the technology of encasing bioactive compounds in solid, liquid or gaseous matrices, which can be released under particular circumstances with a controlled rate”. Recent research has shown different material properties and reactions at the nanoscale, which resulted in the evolution of research towards nanoencapsulation. These nanoencapsulates provide higher bioavailability, solubility and permeation, and better interaction due to a higher surface area. They also allow for targeted release and protection against process conditions and environmental stresses [3].

Nanoencapsulation is, by definition, the encapsulation of materials in the nanoscale, i.e., below 1 μm in size. The regulatory authorities (Food and Drug Administration (FDA) in the United States of America, European Union (EU) commission in Europe) have, in recent years, introduced regulatory definitions for nanoparticles (NPs) and nanomaterials. These definitions are not uniform, and are enforced differently across different sectors. When it comes to food applications, the European Food Safety Authority (EFSA) defines NPs as an engineered nanomaterial, with a size between 1 and 100 nm. If such a material is present in a certain product, it must go through an extensive and thorough risk assessment to determine if it is nonhazardous [4,5,6]. In terms of pharmaceutical science, particles up to 1000 nm can still be classified as nanoparticles due to their unique physicochemical properties compared to those of bulk materials [7].

Some of these materials, such as fish oils, essential oils or some other nutraceuticals, have particularly unpleasant flavors that limit their addition to food formulations. Nanoencapsulation may be useful in masking unwanted flavors in foods [8].

Working with plant-derived natural compounds poses some challenges, such as low stability and low bioavailability, chemical degradation during storage, high molecular weight/size, poor plasma membrane permeability and sensitivity to ultraviolet light and oxygen. Nanocarrier systems may be a useful way to improve the delivery of bioactive molecules which are present in plants, such as antioxidants, vitamins, fatty acids, minerals, phenolic compounds, carotenoids and essential oils [9]. Many of these compounds are hydrophobic or poorly soluble, which is another problem that can be addressed using nanoencapsulation [10].

Lipid-based techniques have been extensively studied and developed for a variety of applications. They allow for the entrapment of bioactive compounds with diverse solubilities, separate or simultaneous encapsulation of hydrophobic and hydrophilic compounds, scalable production and targetability in the human body or food matrices [11]. Nanocarriers composed of other building blocks—namely carbohydrates or proteins—pose problems in terms of industrial scaling up due to the use of intricate chemical and heat processes that cannot be completely monitored. Additionally, an availability of digestible lipids aids in the intestinal absorption of bioactive compounds [12,13].

In this review, various lipid-based nanoencapsulation techniques are described. Several studies that have been done on each technique from 2007 onward are presented. In these research papers, both natural and synthetic compounds were encapsulated. The natural compounds can be extracted from different types of fauna and flora, such as animal tissue, plants, macro- and micro- algae, etc. Nanoencapsulation will allow us to overcome two of the most important barriers in the organism. On the one hand, it will protect the drug against gastrointestinal digestion, and on the other, it will make it possible to efficiently deliver the drug across the BBB. 

## 2. Gastrointestinal Absorption of Nanoparticles

Lipid digestion takes place in the gastrointestinal tract (GIT). It is a complex combination of biochemical and physiochemical processes. Absorption can happen either by enterocyte-based transport or via the intestinal lymphatic system. The lymphatic system comprises an extensive network throughout the body, and thus allows for the avoidance of first-pass metabolism and the targeting of certain diseases known to spread through the lymphatics, such as lymphomas and HIV [14]. Some orally administered drugs display low bioavailability due to a presystemic, or first-pass, metabolism; this can happen in both the intestinal mucosa and the liver. Any foreign molecule absorbed in the small or large intestine must pass through the liver via the hepatic portal vein before gaining access to other parts of the body. This occurs because the drug absorbed in the GIT enters the portal circulation before entering the systemic circulation. Via the portal circulation, it enters the liver, where it undergoes extensive biotransformation, leading to a decrease in the drug concentration which is able to reach the bloodstream [15].

Digestion starts in the mouth, with food being mechanically broken down to smaller pieces. Further mechanical processes lead to the formation of a coarse emulsion. The main digestion of lipids is carried out by pancreatic lipases within the duodenum, especially for liposomes and phytosomes, because gastric lipases have no activity on phospholipids [16].

Lipases adsorb onto the surface of the formed emulsions and hydrolyze the outer ester bonds of the triglycerides, leading to the formation of a monoglyceride and two free fatty acids [17]. These are solubilized by bile salts, biliary-derived phospholipids and cholesterol, and generate a series of colloidal structures such as micelles and unilamellar and multilamellar vesicles. The encapsulated compound is resolubilized into these structures, substantially increasing the drug solubilization capacity of lipid-based nanoparticles compared to that of the free drug [14,18]. The extremely fine particle size of the micelles allows them to permeate and be absorbed between cells through membrane internalization and lymphatic transportation [16].

Iwanaga et al. [19] studied the effects of coating the surface of liposomes on the gastrointestinal transit of insulin in male Wistar rats. Liposomes were coated with poly(ethylene glycol) (PEG) or the sugar chain of mucin, with increased resistance to bile salts. The authors observed that the coating increased their overall stability in the GIT. PEG coated liposomes are retained longer in the small intestine and mucin coated liposomes are retained longer in the stomach. The PEG coated liposomes displayed a two-fold increase in mean retention time compared to mucin coated and uncoated ones. Mucin coated liposomes displayed the highest recovery ratio of insulin (80.71 ± 5.29%), while PEG-coated and uncoated liposomes had lower ratios (64.54 ± 1.19 and 52.57 ± 4.67%, respectively). These results showed that surface coating of nanoparticles can lead to an increase in the bioavailability of orally delivered peptides.

Liu et al. [20] studied the stability of liposomes and nanoliposomes prepared from milk and soybean-derived phospholipids during digestion. During digestion in simulated gastric fluid, there was no significant change in the diameter of the NPs. However, for simulated intestinal fluid, there was a marked increase in diameter. The structure of the liposomes and nanoliposomes was observed with confocal laser scanning microscopy (CLSM) before and after digestion, and a clear degradation was observed. Lastly, the leakage of calcein from the NPs was assessed in simulated gastric fluid. The soybean liposomes and nanoliposomes released 8 and 12% calcein, respectively, while the milk liposomes and nanoliposomes released 6 and 8% calcein, respectively. For simulated intestinal fluid, significantly higher releases were visualized, with all formulations displaying significant calcein release during the first 20 min, and a gradually increasing release rate to about 75% after 240 min.

Liu et al. [21] developed a polyelectrolyte delivery system based on alginate and chitosan which was used to coat the surface of nanoliposomes. The coating increased the mean diameter of the NPs from 89.3 ± 11.8 nm to 330.6 ± 37.3 nm, the polydispersity index from 0.26 ± 0.05 to 0.37 ± 0.12 and the zeta potential decreased from −6.34 ± 0.62 to −15.79 ± 0.70 mV. The changes in simulated gastric fluid were negligible, while in simulated intestinal fluid, the particle size of the polyelectrolyte delivery system increased from 335 to 620 nm over the first 15 min and decreased to 530 nm at the end of digestion. This can be explained by a decrease in the number of cationic groups, which results in a decrease in electrostatic interactions between alginate and chitosan, with the medium being able to enter the particles and increase particle size. A subsequent decrease can be explained by an affinity of chitosan for ions in bile salts. The coating also resulted in a lower amount of medium-chain fatty acids released in simulated gastric fluid, i.e., 13.8% for uncoated nanoliposomes and 13.1% for the polyelectrolyte delivery system after 15 min, and 29.8% and 20.4% after 120 min, respectively. In simulated intestinal fluid, the release rate after 120 min was 79.5% for uncoated nanoliposomes and 56.9% for the polyelectrolyte delivery system.

The exchange of molecules between the blood and the brain is limited by the blood-brain barrier (BBB) and controlled by endothelial transport systems in the brain [22].

## 3. Blood-Brain Barrier

The blood-brain barrier is a complex cellular network, responsible for limiting the free diffusion and penetration of unwanted drugs or other compounds from the bloodstream to the brain while providing the needed nutrients for proper brain function [23,24].

**Figure 1:**
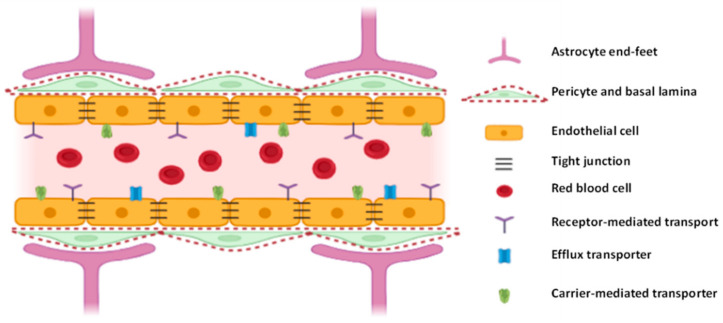
Key components of the blood-brain barrier. Adapted from [2].

It is composed by three main components: endothelial cells, pericytes and astrocytes, along with some other components, such as basement membranes and microglia, represented in Figure 1. The endothelial cells (ECs) lining the cerebral blood vessels are the basic building blocks of the BBB, connected by tight, adherens and gap junctions. This grants them great resistance (i.e., transendothelial electrical resistance (TEER) of 100–150 Ω.cm2 in human cells [25]) and restricts transport across the barrier. They are also fundamentally different from other ECs located in different parts of the body, containing as much as six times more mitochondria per capillary section, which is thought to be necessary to provide the energy required for active transport across the BBB. The pericytes envelop the ECs, determining the permeability of the BBB and some of its functions, such as the strengthening of tight junctions, specific gene expression and polarization of astrocytes end-feet. The pericytes play a key role in the regulation of development and maintenance of the BBB. Astrocytes completely cover the cerebral blood cells with their end-feet and contain several proteins which are indispensable for the proper functioning of the BBB. They also link up the ECs with microglia and neurons. The basement membranes, a complex layer of extracellular matrix proteins, provide an anchor for the cellular components of the BBB. The microglia are monocyte lineage cells, with two main functions: immune defense and CNS maintenance. The tight junctions, formed by several transmembrane and cytoplasmic proteins, are mainly responsible for regulating endothelium permeability, cell polarity and leukocyte migration. The adherens junctions are formed by transmembrane glycoproteins, i.e., cadherins, and allow for the formation of the tight junctions and the maintenance of the BBB characteristics. The gap junctions are located between the other two junctions and allow for the transfer of ions and small molecules between ECs, an essential step for maintaining tissue homeostasis. Gap junctions also transduce metabolic signals and regulate BBB permeability by interacting with scaffolding proteins [2,22,26,27].

The BBB is integral to the proper functioning of the brain, serving a number of functions, namely, brain nutrition, regulation of ionic composition, regulating the entry of macromolecules into the brain, protection against neurotoxins and regulation of neurotransmitters [27].

It has been estimated that the BBB can exclude up to 98% of the small-molecule available drugs from entry into the brain, and that only approximately 0.1% of intravenously administered therapeutic antibodies enter the brain. This means that a significantly higher concentration must be administered, which can lead to systemic toxicity [26].

Despite the BBB acting as a barrier for the passing of molecules between the blood and the brain parenchyma, there are a few transport routes available to allow for the delivery of molecules which are essential to the maintenance of brain homeostasis. These include diffusional transport (paracellular and transcellular transcytosis), transporter proteins mediated transcytosis, receptor-mediated transcytosis, adsorptive mediated transcytosis and cell-mediated transcytosis [22,28]. However, several factors may contribute to BBB integrity breakdown, namely, junctional proteins, proteins at the basement membranes of the BBB, inflammatory mediators, free radicals, vascular endothelial growth factor, matrix metalloproteinases, microRNAs, anesthetic agents, etc. [29]. BBB breakdown is characterized by pericyte and endothelial degeneration with loss of tight and adherens junctions and increased bulk flow transcytosis. The increase in BBB permeability induces (1) the accumulation of neurotoxic factors, (2) impaired glucose transport and impaired P-glycoprotein 1 function, (3) red blood cell extravasation, which leads to ROS generation, (4) inflammatory and immune responses with microglial and astrocytes activation and increased production of pro-inflammatory cytokines and chemokines, and (5) microbial neuroinfections. These impairments culminate in neuronal injury, synaptic dysfunction, loss of neurons and of brain connectivity, thus, causing neurodegeneration [30]. Disruption of BBB integrity and functions is involved in a growing list of brain disorders, such as Alzheimer’s, Parkinson’s, Huntington’s, amyotrophic lateral sclerosis, migraine, traumatic brain injury, intracerebral hemorrhage, multiple sclerosis, Japanese encephalitis and autoimmune encephalomyelitis [29].

**Figure 2:**
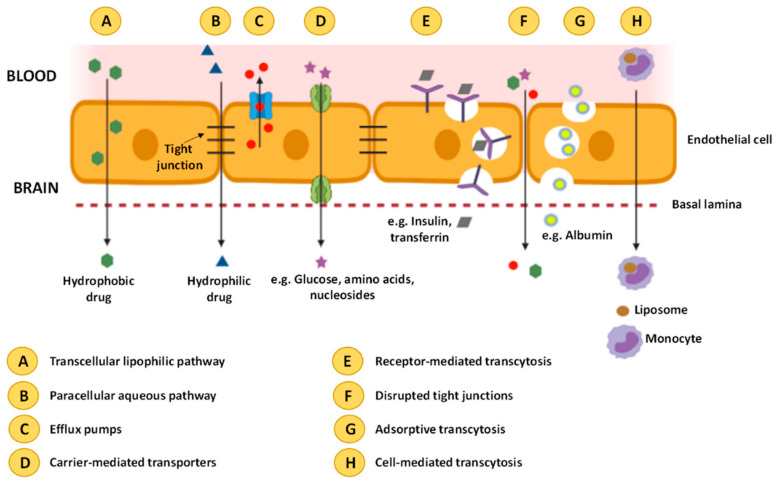
Transport mechanisms across the blood-brain barrier. Adapted from [28].

In a healthy BBB, transcellular diffusion (Figure 2A) is the diffusion of solute particles through the ECs. Particles transported through this route are small lipid soluble substances that penetrate through the cells by dissolving in their lipid plasma. The driving force for this transport is the same as for paracellular diffusion. Paracellular diffusion (Figure 2B) is the passage of small water-soluble molecules (molecular weight <500 Da) through a space between two ECs. The negative concentration gradient from blood to brain is the driving force for this transport. Both types of transport are nonspecific approaches [22,28].

Efflux pumps (Figure 2C) are a set of proteins, including P-glycoproteins and multidrug resistant proteins, which are responsible for limiting the accumulation of various potentially toxic molecules, and afterwards, for expelling these molecules from the brain. These proteins are a limiting factor for the delivery of bioactive compounds to the brain [22,28].

An active transport mechanism is the carrier protein mediated approach (Figure 2D), with the use of glucose transporter isoform (GLUT-1), large amino acid transporter (LAT), or others. Glucose or the amino acids bind with the protein at the blood side of the BBB and a subsequent conformational change allows for transport to the brain, from a higher to a lower concentration. ATP can provide energy required for the process to be performed in the opposite direction, i.e., flowing from a lower to a higher concentration. The application of this mechanism for drug transport is limited by the fact that these transporter proteins carry only specific substances (glucose for GLUT-1, amino acids for LAT) [22,28].

Receptor mediated transcytosis (RMT) (Figure 2E) relies on receptors present on the cell surface, and is widely used nowadays for NP drug delivery. This type of transport relies on endocytosis, where the substance binds with the receptor and an intracellular vesicle is formed through membrane invagination. The most targeted receptors in RMT are transferrin, lactoferrin, insulin, diphtheria toxin and low-density lipoprotein receptors. The disruption of the tight junctions (Figure 2F) can also increase the BBB permeability, increasing the permeability of several compounds to the CNS. [22,28]. 

The adsorptive mediated transcytosis (AMT) (Figure 2G) method is used for the transport of charged particles by taking advantage of the electrostatic interactions between positively charged drug carriers and negatively charged microdomains on the cytoplasmatic membrane surface. This transport method has lower affinity but higher capacity than RMT [22,28].

Cell-mediated transcytosis (Figure 2H) relies on immune cells (neutrophils, monocytes and macrophages) with the ability to cross the BBB in both healthy and disease conditions. In this technique, drugs are encapsulated in a liposome, which are, in turn, absorbed by immune cells that cross the BBB and migrate towards the inflammation sites in the brain. This is a more recent approach, and, unlike the previously mentioned methods which can only carry molecules with specific properties, cell-mediated transcytosis can be used for any type of molecule [22,28].

Due to the tightness of the BBB, much research has been done in the last few years to develop ways to effectively carry drugs across the barrier. Of these, lipid-based techniques are the most studied, with citicoline liposomes being studied for the treatment of cerebral ischemia [31], neuroprotection by quercetin [32] and epilepsy treatment by phenytoin [33]. Phytosomes with *Ginkgo biloba* L. extract were also developed to protect the brain and vascular system of people over 50 years of age [34]. Another technique broadly researched is NLCs, e.g., for anticancer activity [35] and chemotherapy with drugs encapsulated in SLNs [36].

Lipid-based nanoencapsulation techniques are the most used techniques for the targeted delivery of drugs across the BBB.

Hu et al. [37] developed glutathione PEGylated nanoliposomes based on either egg yolk or hydrogenated soy phosphatidylcholine to deliver methotrexate to the brain. Compared to the free drug, the liposomes resulted in a concentration in the plasma of rats which was 717–4330 times higher. However, glutathione does not always result in an increase in brain uptake. Although in hydrogenated soy nanoliposomes, glutathione-PEG coating resulted in a four-fold increase in brain uptake when compared to simple PEG coating, in egg yolk nanoliposomes, it did not show any meaningful increase.

In a study by Chen et al. [38], nanoliposomes encapsulating α-mangostin, a potential candidate for the treatment of Alzheimer’s disease, were modified by transferrin. The uncoated NPs and the transferrin coated NPs displayed an average particle size, polydispersity index and zeta potential of 188 ± 5.29 and 196.3 ± 7.09 nm, 0.201 ± 0.019 and 0.211 ± 0.034 and −17.85 ± 6.0 and −22.23 ± 2.87 mV, respectively. In vitro studies, in which a membrane with a TEER of 210 Ω/cm^2^ was used, showed that the liposomes were able to penetrate the BBB without significant changes to the morphology of the NPs, and in vivo studies demonstrated an improvement in the brain delivery of α-mangostin, increasing the half-life from 0.76 to 0.82 h and the mean residence time from 0.55 to 0.77 h.

## 4. Delivery Routes for Neuroprotective Drugs

Among the advantages of oral administration, one can mention its convenience, acceptance by patients, variety of dosage forms available and the fact that it is noninvasive. However, it is prone to GIT degradation, hepatic first-pass metabolism, fluctuant bioavailability, delayed onset of action, pharmacokinetic variability and is not suitable for emergencies [39,40]. Besides oral administration, other delivery routes can be used to transport neuroprotective drugs into the brain. Intracerebroventricular (ICV) injection allows the delivery of drugs into the brain through the cerebrospinal fluid, and intravenous (IV) and intramuscular administration introduce different therapeutics into the circulatory system, leading to systemic delivery to the CNS. The primary benefit of ICV is that it can effectively bypass the BBB [41]. Compared with oral administration, IV is characterized by fast onset of action, high bioavailability, avoidance of absorption and hepatic first-pass metabolism, and is useful in emergencies. However, IV injections are painful and invasive [39,40]. The intramuscular route is useful when IV access is lacking; additionally, it offers fast onset of the desired therapeutic effect. As for IV, is painful and invasive [39,40]. Intranasal delivery can provide an unparalleled opportunity to deliver drugs to the CNS by direct access to the brain through the olfactory and trigeminal nerve pathways. In this way, it allows drugs to circumvent not only presystemic gastrointestinal and hepatic elimination, but also to bypass the BBB. Intranasal delivery is associated with several advantages over other brain delivery routes, e.g., enhanced safety, increased patient compliance, ease of administration, rapid onset of action, and minimum systemic exposure. On the other hand, it also presents some disadvantages, namely, drugs may be rapidly eliminated from the nasal cavity due to mucociliary clearance, high molecular weight drugs are relatively less permeable across the nasal mucosa, drugs may also undergo degradation by enzymes present in the nasal mucosa, short retention times, restrictions imposed by the geometry of the nasal cavity and lack of targeting specificity to the affected area of the brain. Thus, nose-to-brain delivery has mostly been restricted to the administration of extremely potent molecules effective in the brain at concentrations in the nanomolar range or at even lower [42].

### 4.1. Intracerebroventricular Delivery Route of Lipid-Based Nanoparticles

Rungta et al. [43] used small interfering RNA (siRNA) in lipid nanoparticles (LNPs) to efficiently silence the gene GRIN1 in hippocampal neuronal cultures and in in vivo through intracortical or ICV injections. GRIN1 encodes the GluN1 subunit of the NMDA receptor. The compositions of LNPs consist of 3-(dimethylamino)propyl(12Z,15Z)-3-[(9Z,12Z)-octadeca-9,12-dien-1-yl]henicosa-12,15-dienoate (DMAP-BLP)/distearoylphosphatidylcholine (DSPC)/cholesterol/PEG-DMG (50/10/37.5/1.5). LNP-siRNA systems exhibited efficient gene silencing properties in neurons both in vitro and in vivo, without inducing significant toxicity. In vivo, intracortical or ICV siRNA-LNP injections resulted in knockdown of the target gene in discrete regions around the injection site and in more widespread areas in the case of ICV injections. The LNP-siRNA approach has been shown to be an effective alternative to other in vivo transfection vectors presently in use, such as viral delivery, since this requires the time-consuming construction of virus vectors, potentially causes immune responses and raises safety concerns. 

### 4.2. Intravenous and Intramuscular Administration of Lipid-Based Nanoparticles

Some neurotransmitters have demonstrated the ability to cross the BBB. Ma et al. [44] synthesized a series of lipidized neurotransmitter derivatives, called neurotransmitter-lipidoids, and observed that those composed of tryptamine could effectively cross the BBB, while those based on phenethylamine and phenylethanolamine could not. The tryptamine-lipidoids were doped with LNPs, and the resulting lipid nanoparticles also gained the ability to cross the BBB. Using these carriers, the authors successfully delivered the antifungal amphotericin B, antisense oligonucleotides (ASOs) against tau, and genome-editing fusion protein (−27)GFP-Cre recombinase into mouse brain via systemic IV injection. This was a great accomplishment, since the BBB is impermeable to these drugs, limiting their application for the treatment of CNS fungal infections and neurodegenerative disorders [44].

Andrographolide is a natural diterpenoid that displays protection against oxidative stress mediated neurotoxicity, inflammation-mediated neurodegeneration and cerebral ischemia. Despite its biological potential, andrographolide shows low bioavailability, poor water solubility and high chemical and metabolic instability. Graverini et al. [45] prepared SLNs to deliver andrographolide into the brain using Compritol 888 ATO as a solid lipid and Brij 78 as a surfactant. Using in vitro BBB permeation tests, the authors showed that SLNs improved the permeability of andrographolide compared to that of the free compound. Afterwards, fluorescent nanoparticles were prepared for in vivo tests in healthy rats. After IV administration, fluorescent SLN were detected in brain parenchyma outside the vascular bed, confirming their ability to cross the BBB.

Curcumin bioavailability in brain after oral administration is very low, with less than 1% of the administered dose being systemically available. Polysorbate 80 (45.45%) and soy lecithin (0.58%) SLNs were prepared to deliver curcumin to the Balb/c mice through IV and oral administration [46]. The concentration of curcumin in brain after administration of curcumin-SLNs was significantly higher than for free curcumin administered through oral and IV injection, with IV administration being the most efficient delivery route. The AUC_brain_ for curcumin-SLNs administered through IV injection was 30.82 times that of the free compound, while for oral administration, it was 16.4 times more.

Koshkina et al. [47] evaluated the concentration of camptothecin, a natural topoisomerase I inhibitor, in five organs (lungs, blood, liver, kidney, brain) and tumors of mice after application of camptothecin liposome aerosol at a dose of 81 µg/Kg. They compared this treatment with a previous study in which oral, IV and intramuscular routes were also tested, by using camptothecin dispersed as fine emulsion in Intralipid 20 at a dose of 4 mg/Kg [48]. After 30 min, the camptothecin concentration in brain was 61, 37, 12 and 27 ng/g tissue following aerosol, oral, IV and intramuscular administration, respectively. 

### 4.3. Intranasal Delivery of Lipid-Based Nanoparticles

Nose-to-brain transport occurs mainly via the systemic, olfactory and trigeminal nerve pathways, which differ concerning the drug absorption site and the amount of time required for absorption to occur [42].

Borneol is a well-known compound in traditional Chinese medicine; it is used to guide drugs to the brain. Since *Pueraria* flavones (PTF) exhibit low bioavailability and difficulties in reaching the brain, three different SLN formulations were prepared to deliver these flavonoids into the brain through the nasal cavity [49]. PTF-borneol-stearic acid-SLNs, PTF-borneol-SLNs and PTF-SLNs displayed similar sizes (between 154.2 ± 1.1 and 165.2 ± 0.9 nm) and drug loading capacity (4.60 ± 0.01 and 4.81 ± 0.07%), and were all spherical with a uniform size (polydispersity index between 0.12 ± 0.01 and 0.29 ± 0.05). In vitro release studies using the dialysis bag method demonstrated that about 80% of PTF was released from the three SLNs after a 12-h incubation period. In Caco-2 cell line, which is derived from intestinal mucosa and can be used as a model of the nasal mucosa epithelia, the uptake of PTF-borneol-stearic acid-SLNs and PTF-borneol-SLNs by the cells was higher than that of PTF-SLNs at 2 h. For in vivo studies, a fluorescent probe (coumarin-6) was loaded into SLNs to evaluate the brain delivery properties of SLNs after intranasal delivery to Sprague-Dawley rats. A lower fluorescence signal was observed in coumarin-6-borneol-SLNs group compared with coumarin-6-borneol-stearic acid-SLNs in the brain, while the opposite was observed for the olfactory bulb area. The results highlighted the better targeting effect of the borneol-stearic acid-modified SLNs, with the area under the curve (AUC) and C_max_ of PTF by these SLNs being about 5.95- and 5.98-fold greater compared with those of PTF-borneol-SLNs. The in vivo results also proved that PTF-borneol-stearic acid-SLNs is mainly delivered through the trigeminal pathway, while PTF-borneol-SLNs entered into the brain mainly through the olfactory pathway. 

## 5. Lipid-Based Nanoencapsulation Techniques

There are several types of nanoformulations which allow for the encapsulation of bioactive compounds. Most are based on different building blocks, namely carbohydrates, proteins or lipids. These have usually similar characteristics, namely, being biocompatible and biodegradable, but differ in other capacities, such as the production method, particle size, structure, morphology, pharmacodynamics and therapeutic properties [50].

The advantages of lipid-based nanoencapsulation are the possibility of industrial production, higher encapsulation efficiency (%EE) and low toxicity. In addition, considering that compounds usually encapsulated, such as flavonoids, polyphenols, carotenoids and fatty acids, have different polarities, lipid-based NPs also allow for the separate or simultaneous encapsulation of both hydrophilic and hydrophobic substances, due to their amphiphilic behavior. The use of digestible lipids facilitates the intestinal absorption of bioactives, since they solubilize and carry the hydrophilic compounds [11,12].

The main types of lipid-based nanoencapsulation techniques are nanoemulsions (NEs), nanoliposomes and nanophytosomes, and nanostructured lipid carriers (NLCs), which will be explained in the following chapter.

### 5.1. Encapsulation by Nanoemulsions

**Figure 3:**
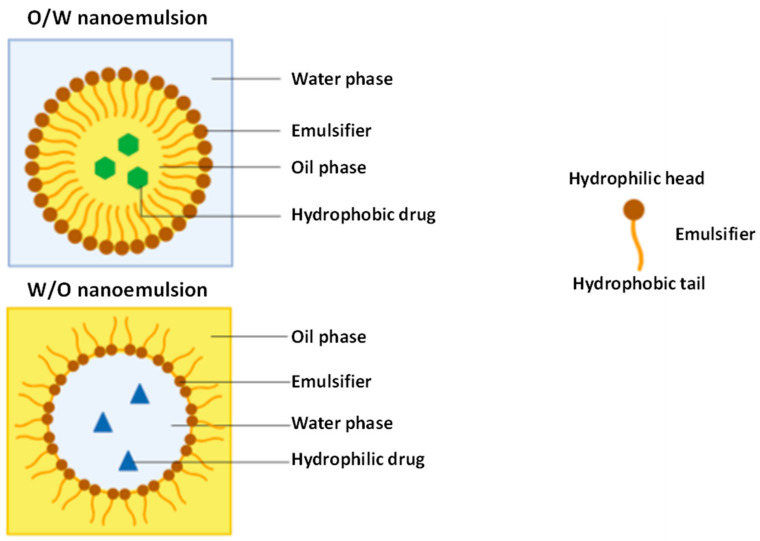
Structure of an O/W nanoemulsion (top) and W/O nanoemulsion (bottom). Adapted from [58,59].

NEs are isotropic colloidal systems composed of two immiscible liquids, stabilized by amphiphilic surfactant molecules. NEs can be in the form of an emulsion of oil-in-water (O/W) or water-in-oil (W/O) [51]. Both types of NEs are exemplified in Figure 3. The mean droplet diameter covers a size of 50–200 nm in a transparent nanoemulsion and up to 500 nm when it has a milky appearance [52]. Double NEs have also been developed, either as W/O/W or O/W/O [50].

Unlike microemulsions, which are transparent and thermodynamically stable, NEs cannot be formed spontaneously, as they are nonequilibrium systems. This means that NEs have a higher solubilization capacity for lipophilic drugs and better resistance toward droplet collisions, which confers great kinetic colloidal stability upon them [53]. 

They are thermodynamically unstable, since their free energy of formation is greater than that of their separated states. Despite this, due to their nanosized droplets, NEs have long-term physical stability without apparent flocculation during storage. NEs are sometimes referred to as “Approaching Thermodynamic Stability” [54,55].

NEs are widely used in the food and nutraceutical industries [4] and for drug delivery in the treatment of neurological disorders such as Alzheimer’s, Parkinson’s and Prion’s [56] due to their various advantages, such as a very small droplet size that causes a large reduction in gravity force, which may allow Brownian motion to be enough to overcome gravity. This means that no sedimentation will occur in storage. The small droplet size also prevents flocculation of the drops, which enables the system to remain dispersed without the need for separation [57]. The small droplets are nondeformable, which prevents surface fluctuations. Also, a significant surfactant film thickness, relative to the droplet radius, prevents thinning or disruption of the liquid film between the droplets. Nanoemulsions have a large surface area, which makes them effective in delivering active ingredients through the skin. They require a considerably lower concentration of surfactant when compared to microemulsions, i.e., in the region of 20% or higher. For nanoemulsions, surfactant concentrations in the range of 5–10% may be sufficient [54].

Although they have many advantages, there are several factors that limit their application: the preparation may require special application techniques, such as high-pressure homogenizers or ultrasounds; they are expensive to produce, due to the need for expensive equipment and high concentrations of emulsifiers; there is a lack of understanding of the mechanism of production of submicron droplets or the role of surfactants and cosurfactants; the benefits of nanoemulsions over conventional macroemulsion systems have not been adequately demonstrated, and there is a lack of knowledge regarding the mechanisms that affect and cause problems during their production (such as the Ostwald ripening, i.e., the oil diffusion in the aqueous phase) [54]. 

Teo et al. [60] formulated lutein NEs stabilized by whey protein isolate by two different methods, i.e., emulsification and solvent evaporation, which resulted in encapsulation efficiencies of 86.3 and 80.7% and particle sizes of 147.3 and 68.8 nm, respectively. The NEs with smaller particle size were optically translucent, while the conventional emulsions were opaque due to larger particles being capable of scattering lighter. The developed NEs were relatively stable for 20 days at 5, 20 and 40 °C, with no increase in particle size, although some loss of lutein content was observed, especially at 40 °C. NEs displayed higher lutein loss than conventional NEs. An MTT assay was used to prove that the formulated NEs were nontoxic to cells. NEs also displayed a higher cellular uptake of lutein in comparison to conventional emulsions.

Vishwanathan et al. [61] performed a preclinical trial, comparing the bioavailability of lutein administered in a supplement or through NEs. The NEs displayed a mean diameter of 150 nm and were administered in two different ways: 6 and 2 mg/day. Although the actual concentration of lutein was 10 and 40% lower compared to the serum, the NEs resulted in a 31 and 28% increase in lutein serum concentrations when compared to the supplements.

The typical main components involved in the formulation of NEs are oil, a surfactant and a cosurfactant, a surfactant mixture (S_mix_) and an aqueous phase at appropriate ratios. Nevertheless, multiple other ingredients can be used in both the internal and external phases [59].

#### 5.1.1. Oil

Several types of oils can be used in the formulation of NEs, depending on which drug is to be incorporated. Therefore, the solubility in oil of drugs is often tested. The oil represents one of the most important components of NEs, given its ability to solubilize lipophilic drugs, but also to improve the fraction of drug transported through the intestinal lymphatic system, thereby increasing GIT drug absorption [59].

Hydrolyzed vegetable oils, medium-chain and modified long triglycerides are usually used in NE formulation, generally containing 5 to 20% oil/lipids in the case of O/W NEs, although this number can go as high as 70%. Edible oils are not usually selected for NE formulation due to their poor ability to dissolve large amounts of lipophilic drugs [59,62].

Arora et al.[63] screened four oils (Capmul MCM, soybean oil, grape seed oil and vitamin E) for the formulation of a tetrabenazine NE. The calculated solubilities for the different oils were 2.50 ± 0.26, 1.00 ± 0.09, 1.50 ± 0.17 and 0.50 ± 0.04 mg/mL, respectively, with Capmul MCM being selected as the optimum oil for NE formulation.

Đorđević et al. [64] attempted to solubilize the antipsychotic risperidone in medium-chain triglycerides (MCT) and soybean oil, as well as their mixtures, but could not achieve the target concentration of 1 mg/g risperidone in the NE. Lecithin was added to the oil/mixture of oils, and an increase in temperature was applied, without success. The target concentration was achieved by dissolving risperidone in a cosolvent, benzyl alcohol, with the final oil ratio being 4:1 *w*/*w* MCT: soybean oil.

#### 5.1.2. Surfactant

Surfactants are amphiphilic molecules used to stabilize the NEs by reducing the interfacial tension between the oil and the aqueous phases. They adsorb onto oil droplets, forming a flexible film that can deform around them. Surfactants consist of a hydrophilic head and a hydrophobic tail and act as emulsifiers in the production of the NEs. A wide range of surfactants can be used in the formulation of NEs [62,65].

Haider et al. [66] screened different surfactants (Cremophor EL, Cremophor RH 40, Capryol 90, Labrafil M, Labrasol and Tween 80) for the production of rivastigmine hydrochloride NEs. Tween 80 (45 ± 2 mg/mL) and Capryol 90 (35 ± 2 mg/mL) displayed the highest solubility rates, with Tween 80 being chosen as the surfactant.

Shu et al. [67] developed NEs of astaxanthin, a carotenoid found in several microorganisms and aquatic animals such as algae, trout, krill, crayfish and salmon. The formulated particles were stabilized by the addition of natural surfactants, ginseng saponins, which are well known for their neuroprotective effects. The developed particles displayed an average diameter of 125 nm, thermal stability between 30 and 90 °C, good stability during 15 days of storage and an increase in emulsion concentration. Additionally, homogenization pressure resulted in a decrease in particle size. However, the NEs were unstable in acidic conditions (pH 3–6) and high salt levels (> 25 mM, NaCl.).

Khalid et al. [68] also developed astaxanthin NEs using two different surfactants, i.e., modified lecithin (ML) and sodium caseinate (SC), obtaining diameters of 163 and 144 nm, respectively. In SC-stabilized NEs, with pH 4, phase separation occurred, while the mean diameter suffered only slight variations in ML-stabilized NEs. As for ionic strength, at concentrations above 500 mM NaCl, there was no increase in mean diameter in SC-stabilized NEs, while in concentrations above 300 mM NaCl, there was an increase in mean diameter and oiling off was observed in ML-stabilized NEs. Freeze-thaw cycles were also applied, with mean diameters increasing from 136 to 403 nm after four cycles in ML-stabilized NEs, while in SC-stabilized NEs, the mean diameters increased from 115 to 150 nm. High temperature treatments were also applied, with both NEs showing no significant growth of droplet size up to 90 °C, while at 120 °C, SC-stabilized NEs displayed significant droplet growth, which did not happen in ML-stabilized NEs. As for bioaccessibility, SC-stabilized NEs displayed only 6%, while ML-stabilized NEs displayed values above 32%.

Weigel et al. [69] tested the influence of different surfactants (quillaja saponin, Tween 80, whey protein isolate, and sodium caseinate) in the development of lutein NEs. The first three surfactants resulted in NEs with mean diameter sizes in the range of 220 to 250 nm, while the latter resulted in sizes of approximately 600 nm. The Tween 80 stabilized particles displayed a zeta potential of −9 mV, which resulted in low stability NEs. The other three emulsions displayed zeta potentials between −42 and −62 mV. Emulsion stability was assessed through storage at 45 °C for 10 days and color degrading analysis. The quillaja saponin and whey protein isolate NEs displayed no creaming and oiling off, while the other two emulsions displayed the opposite, indicating a lack of robustness of the interfacial layers in stabilizing the system. Based on all parameters analyzed, quillaja saponin was selected as the optimum surfactant. The authors also analyzed the influence of the inclusion of antioxidants in the formulation on the stability of the formulated NEs. The antioxidants introduced were ascorbic acid, catechin, alpha tocopherol, ascorbic acid palmitate and EDTA. The antioxidants had no effect on the physical stability of the NEs, but, except for ascorbic acid, they promoted carotenoid degradation. Only ascorbic acid inhibited some color fading during storage.

Frede et al. [70] assessed the use of different surfactants in the formulation of lutein NEs. The analyzed surfactants were β-lactoglobulin, β-lactoglobulin/lecithin, Biozate 1, Biozate 1/lecithin, Tween 20 and Tween 20/lecithin. The surface area mean diameters were measured, with the Tween 20 formulations displaying the largest oil droplets (540 and 610 nm, respectively), while the other four had droplets with smaller diameters, i.e., between 320 and 260 nm, respectively. All six formulations displayed low physical stability, with the Tween 20 formulations showing creaming, while in the others, aggregation occurred. The β-lactoglobulin and Biozate 1 formulations successfully stabilized lutein inside NEs, which did not happen in the Tween 20 formulations. β-lactoglobulin NEs displayed no cytotoxicity, while Biozate 1 NEs displayed cytotoxicity with increasing Biozate 1 concentration. The authors concluded that β-lactoglobulin, Biozate 1 and its combination with lecithin NEs displayed the most promising results.

#### 5.1.3. Cosurfactant

If a single surfactant is used, the film formed around the droplets is usually highly rigid, leading to the production of NEs over a very limited range of compositions. Therefore, a cosurfactant is usually utilized to efficiently lower the surface tension and confer flexibility upon the interfacial film to guarantee NE formation at higher ranges of compositions. These cosurfactants are usually used at low concentrations due to severe side effects that appear at high concentrations [59,62].

Haider et al. [66] screened four cosurfactants (Captex 200-P, Polyethylene glycol 400, Sorbitan sesquioleate and Transcutol-P) for a rivastigmine hydrochloride NE formulation. Transcutol-P displayed the highest solubility (60 ± 1.5 mg/mL) and was selected as the cosurfactant.

In order to screen the appropriate ratios, a ternary phase diagram is usually constructed to determine the water:oil:Smix which is best suited for the NE formulation [62].

Despite their disadvantages, several studies have been conducted to produce NEs containing neuroprotective drugs. Table 1 reports several studies that have been published regarding the production of NEs.

### 5.2. Encapsulation by Nanoliposomes/Nanophytosomes

Liposomes have been defined as “closed, continuous bilayered structures made mainly of lipid and/or phospholipid molecules” [89]. Liposomes arrange in a polar head group with a long hydrophobic tail. Phospholipid molecules arrange in a bilayer form, with the heads of one layer contacting the outside media and the heads of the other layer surrounding an interior aqueous phase, which confers upon them a general amphiphilic behavior. This allows liposomes to serve as a storage and carrier of drugs with different lipophilicities [90]. 

Nanoscale versions of liposomes are referred to as nanoliposomes. Despite having many similarities to conventional liposomes, they bring the added benefits associated with nanoparticles such as increased surface area and better penetration potential [91].

Liposomes can be divided into different groups according to their size, lamellarity and vesicularity characteristics [92]. These include:

- Unilamellar vesicles (ULV), which contain one single lipidic bilayer and can be a small unilamellar vesicle (SUV, when less than 100 nm) or a large unilamellar vesicle (LUV);
- Multilamellar vesicles, composed of multiple concentric bilayers;
- Multivesicular vesicles (MVV), which are composed of many small nonconcentric vesicles encapsulated within a single lipid bilayer;
- Double bilayer vesicle (DBV), consisting of two bilayer membranes.

Nanoliposomes can be easily adapted to industrial production, due to their broad availability and the relatively low price of their materials, such as crude lecithin. These are a rich source of phospholipids, including phosphatidylcholine (PC), the most used phospholipid [4].

Nanoliposomes are not thermodynamically stable. Researchers have been trying to address their instability, the degradation of the encapsulated materials and the influence of environmental variables, such as the composition, storage temperature, pH and exposure to light and oxygen [93].

Some disadvantages related to the production of nanoliposome are their low encapsulation efficiency (especially for highly water-soluble substances), the wide variation of liposome diameter between batches, difficulties associated with the scale-up, and the use of organic solvents that may impart toxicity upon the products [94]. Despite this, nanoliposomes have been prepared without the use of organic solvents, which makes them nontoxic to cells [95].

Table 2 summarizes several studies in which nanoliposomes were produced to incorporate neuroprotective compounds.

Phytosomes, also referred to in the literature as herbosomes or phyto-phospholipid complexes, are a more enhanced nanoliposomal delivery of bioactive compounds. The term combines “phyto”, the bioactive portion of the complex derived from a plant, and “some”, the cell like part of the complex [117]. Phytosomes are a patented technology, created by the Italian company Indena, that defines them as “a proprietary 100% food-grade delivery system to optimize bioavailability and pharmacokinetic profile of natural actives by formulating them with a dietary ingredient (lecithin).”

The main interaction occurring in phytosomes is between the active compound and phospholipids, with the polar head (the phosphate and ammonium groups) of the phospholipid being bound to the polar groups (the -OH group of phenolic rings) of the bioactive compound. This link is usually described as an H bond, although some researchers have suggested that Van der Waals forces may also play a role [118].

**Figure 4:**
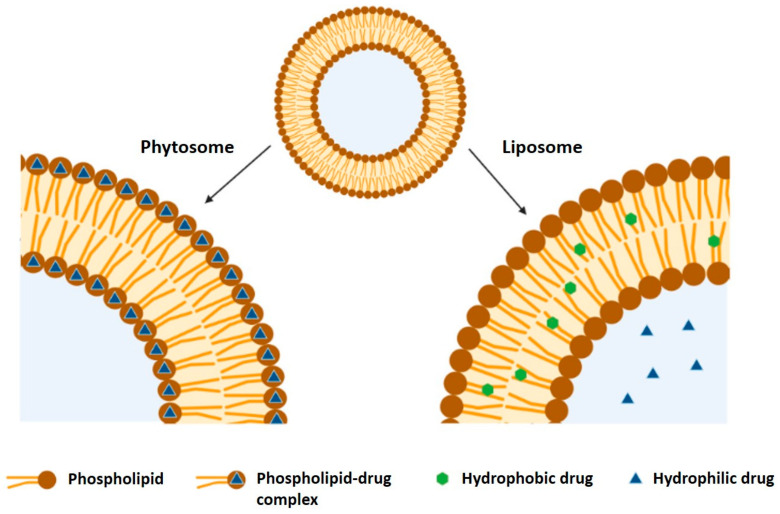
Structure of phytosomes and liposomes. Adapted from [120].

In a water medium, phytosomes assume a micellar shape, forming a liposome-like structure [119]. Despite having similar structures, liposomes and phytosomes have some key differences. In nanoliposomes, hydrophilic compounds are dispersed in the internal space of the particle and lipophilic compounds are dispersed within the lipid bilayer, with no H-bonding taking place, as can be seen in Figure 4. In nanophytosomes, the compounds are chemically linked to the polar head of the phospholipid, being an integral part of the lipid bilayer [120]. Thus, the main difference between both lipid-based NPs is that, in the case of liposomes, the active compound is dissolved in the medium contained in the cavity or in the layers of the membrane, while in phytosomes, it is an integral part of the membrane, i.e., the molecules stabilized through the establishment of chemical bonds to the polar head of the phospholipids. Moreover, the optimum molar ratios of phospholipid to phytoactive compound in phytosomes are 1:1–3:1. But, in liposomes, the amount of phospholipids is approximately five times more than that in phytosomes [121,122]. Due to these characteristics, phytosomes are more stable and allow for higher compound loading capacity than liposomes [123].

The four essential components needed for the production of phytosomes are phospholipids, an active compound, a solvent and an appropriate stoichiometric ratio of active compound to phospholipid [117].

#### 5.2.1. Phospholipids

**Figure 5:**
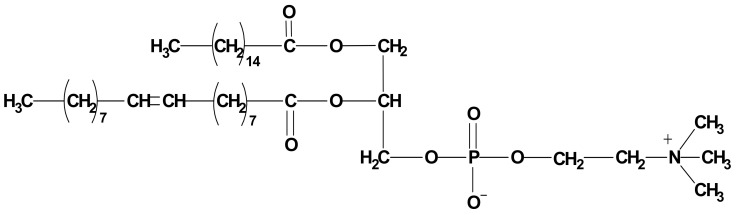
General structure of phosphatidylcholine. Adapted from [118].

Phospholipids can be of natural or synthetic source and are abundant in animal tissues such as egg yolk and bovine brain and plants, such as soy, sunflower and rapeseed [124]. They are amphiphilic molecules, with considerable solubility in both aqueous and lipid mediums. Phospholipids are composed of a glycerol backbone, linked to two fatty acids, with the third linked molecule being a phosphate group. Variations in the head group lead to different phospholipids, with the most used being phosphatidylcholine (PC), phosphatidylserine (PS) and phosphatidylethanolamine (PE) [125]. Of these, PC (Figure 5) is the most widely used. PC possesses two neutral tail groups, the fatty acids, with a polar head group, which contains an oxygen atom with a tendency to gain electrons, and a nitrogen atom that loses electrons. This makes PC miscible in both aqueous and lipid environments [117].

In addition, it has been reported that PC has several beneficial and therapeutic activities, such as hepatoprotective activity, potential as a nutritional supplement for brain health, involvement in membrane fluidity, excellent emulsifying activity, potential for use as a precursor for acetylcholine, the ability to improve the perception of smell and taste, as an aid in the recuperation of fatigue and to nourish skin [124].

#### 5.2.2. Active Compound

The phyto-active compounds utilized are either the active constituents or a standardized extract of a plant. Some natural products lose a part or all their biological activity upon isolation and purification, hence the need for the use of whole plant extracts at times [124]. On the other hand, a purification step of crude extracts can, in some cases, be advantageous when there are compounds in their composition that act antagonistically [126] or compounds that present high toxicity [127] such as pyrrolizidine alkaloids [128], cyanogenic glycosides [129], acetogenins [130], aristolochic acid [131,132], furanocoumarins [133,134] or cardiac glycosides [135], among others. In these cases, nanoencapsulation of purified extracts or bioactive isolated compounds should be carried out in order to find a balance between safety and bioactivity.

Of these compounds, the majority are polyphenols, divided into flavonoids and phenolic compounds, among others. Flavonoids are themselves divided into the following subtypes: flavones (e.g., luteolin), flavonols (e.g., rutin, quercetin), flavanols (e.g., catechin), flavanones (e.g., naringenin, hesperetin), isoflavones (e.g., puerarin, daidzein), proanthocyanidins and anthocyanins (e.g., cyaniding and pelargonidin). Some of these are lypophilic, and are therefore able to diffuse through biological membranes but unable to dissolve in aqueous gastrointestinal fluids, while others have hydrophilic properties, showing affinity for aqueous phases but being unable to surpass biological membranes. Phytosomes can remedy both of these shortcomings, while also protecting these compounds from other factors, such as hydrolysis, photolysis and oxidation [117].

Other compounds have been utilized in the production of phytosomes, such as evodiamine [136], oxymatrine [137], celastrol [138], andrographolide [139], ursodeoxycholic acid [140], nimesulide [141], gymnemic acid [142], emodin [143], oleanolic acid [144], 20(S)-protopanaxadiol [145], berberine [146] and embelin [147]. It has been reported that any compound with π electrons can be used in the formulation of phytosomes.

#### 5.2.3. Solvents

The choice of solvent used in the formulation of phytosomes depends on the solubility of both the phospholipid and the active compound. Initially, mostly aprotic solvents were used, such as dichloromethane [148], dioxane [149], tetrahydrofuran [150] and chloroform [151]. These organic solvents have a high impact on the environment and have been somewhat replaced by protic solvents, such as ethanol [152,153,154] and methanol [155], which are safer. More recently, supercritical fluids (SCF) have shown up as potential replacements [120].

The same active compound can be successfully formulated into phytosomes with the addition of different solvents, which will confer different characteristics upon the phytosome. For example, Babazadeh et al. [156] prepared three rutin nanophytosome formulations (1:1, 1:2 and 1:3 rutin to phospholipid ratio) by dissolving the components in absolute ethanol. All formulations resulted in NPs with mean particle size below 100 nm, and although the formulation with a 1:3 ratio displayed the highest particle size, it also had the highest EE, i.e., approximately 99%, and the highest stability. Therefore, rutin nanophytosomes displayed potential in masking undesirable flavors in foods while maintaining their functionality and increasing stability. Vankudri et al. [157] utilized dichloromethane as a solvent and reported an increase in solubility in both n-octanol and water (approximately 2.7-fold and 25.5-fold, respectively). An increase in the peak concentration of rutin in rat serum (13.2 μg/ mL for the phytosomes against 10.47 μg/ mL for free rutin) was also reported, with a higher concentration maintained for a longer period, which resulted in enhanced therapeutic efficacy for antidiabetic activity. Hooresfand et al. [158] prepared three nanophytosome formulations (1:1, 1:2 and 1:4 rutin to phospholipid ratio) with a mixture of methanol and chloroform (1:4) and assessed the optimal ratio by evaluating the mean particle size during seven days of storage. While on the first day, the 1:1 formulation had the lowest particle size (99 ± 6 nm), followed by the 1:2 and 1:3 ratios (119 ± 7 and 123 ± 10 nm, respectively), after seven days, the particle size of formulations 1:1 and 1:3 increased dramatically (14610 ± 326 and 14651 ± 538 nm, respectively). Only the 1:2 formulation displayed acceptable stability, with a particle size of 403 ± 30 nm. The same method was used to evaluate the addition of cholesterol to the optimal ratio of rutin to phospholipid. Three different ratios were used (1:2:0.2, 1:2:0.5 and 1:2:1 rutin to phospholipid to cholesterol) and particle size was assessed throughout a 21-day storage period. The optimal ratio was discovered to be 1:2:0.2, with a particle size of 164.5 ± 11 nm on the first day and 582.5 ± 43 nm after 21 days. This formulation displayed a –45.2 mV zeta potential, indicating high physical stability and an encapsulation efficiency of 80.4 ± 1.3%.

Maiti et al. [159] developed a phytosome formulation for the encapsulation of rutin by dissolving it in 20 mL dichloromethane, while Tung et al. [160] utilized the same phospholipid and dissolved it in 30 mL dichloromethane. Both experiments had a yield of approximately 88% *w*/*w* when the molar ratio was 1:1, although the latter achieved higher yields when the ratio was 1:2 or 1:4. Despite having very similar yields, the drug content in the phytosomes differed, i.e., 32 and 26%, respectively.

Li et al. [161] published the first paper describing the production of puerarin phytosomes using supercritical carbon dioxide (ScCO_2_) as an antisolvent. The applied method comprised solution enhanced dispersion by supercritical fluids (SEDS), which led to the creation of amorphous, partially agglomerated spheres of about 1 µm in size. The use of ScCO_2_ has a distinct advantage in the sense that it is done in a single step, utilizing a “green” solvent, i.e., CO_2_, which has a critical temperature near room temperature. This diminishes the environmental impact associated with the whole process. Additionally, SEDS makes it possible to control of several parameters, such as the mean particle size and its distribution, surface coating and particle morphology, and allows for easy downstream processing. In this study, several parameters were studied: temperature (30 to 40 °C), pressure (8 to 12 MPa), CO_2_ flow rate (25 to 65 mL/min), proportion of CO_2_ to puerarin solution (1 to 5%) and puerarin concentration in ethanol (60 to 150 mg/mL). The optimal conditions were found to be 35° C, 10 MPa, 45 mL/min CO_2_ flow rate, 1% proportion of CO_2_ to puerarin solution and 100 mg/mL puerarin concentration.

Xia et al. [162] developed lutein proliposomes using a supercritical antisolvent technique (SAS), and studied the influence of different parameters on lutein loading and particle size. The studied parameters were temperature (35 to 55 °C), pressure (8 to 16 MPa) and flow rate of the lutein solution (0.5 to 1.5 mL/min). The optimized formulation was obtained with the following conditions: a temperature of 35 °C, a pressure of 8 MPa and a flow rate of 1 mL/min; this resulted in a lutein loading of 55 mg/g. The liposomes were obtained by hydrating the proliposomes, and an encapsulation efficiency of 90.0% was obtained.

Zhao et al. [163] utilized ScCO_2_ in the formulation of lutein nanoliposomes. The effects of pressure (30 to 300 bar), depressurization rate (20 to 200 bar/min), temperature (40 to 65 °C) and lutein-to-lipid ratio (0.5 to 20 mol %) were assessed. The optimal conditions were found to be a pressure of 300 bar, a depressurization rate of 90 bar/min, a temperature of 50 °C and a lutein-to-lipid ratio of 5%. The authors also proposed a mechanism for the formation of nanoliposomes by the supercritical CO_2_ method. This mechanism comprises four major steps: the presence of phospholipids in bilayers in the aqueous medium, with lutein present in aggregates at ambient temperature and pressure; after pressurization with CO_2_, an equilibrium among CO_2_, water, phospholipids and lutein is formed; the rapid depressurization forces the bilayers and aggregates of phospholipids and lutein to be dispersed in a short-lived monomer state; finally, the phospholipid and lutein rearrange due to hydrophobic and Van der Waals forces, forming the nanoliposomes.

#### 5.2.4. Stoichiometric Ratio of Active Compound to Phospholipid

Different ratios have been studied in the production of phytosomes. It was considered that 1:1 was the ratio that produced the best results, but some studies have disproved this. When different compounds are utilized, the optimal ratios differ.

Kalita and Patwary [164] produced hesperidin phytosomes with different ratios of hesperidin to phospholipid (1:0.5, 1:1, 1:2 and 1:3), and reported that a 1:1 ratio displayed the best results in terms of solubility in distilled water and PBS at pH 2.5 and 7.4, partition coefficient n-octanol/distilled water and n-octanol/PBS pH 7.4 and drug content (92.54 ± 4.01%). The in vitro drug release was also increased from 46.9% after 8 h in free hesperidin to 78.2% by the phytosome formulation.

Telange et al. [149] applied a full factorial design (32) to the production of apigenin phytosomes and studied three different ratios of phyto-active compound to phospholipid (1:1, 1:2 and 1:3) and reaction temperature (40, 50 and 60 °C), with the % of apigenin incorporated as the dependent variable. The optimal values were found to be a 1:2 ratio and 60 °C, which resulted in the highest apigenin incorporation, i.e., 93.26 ± 0.82% *w*/*w*.

Saoji et al. [152] applied a quality by design approach (QbD) to optimize the production of phytosomes containing standardized *Centella asiatica* (L.) Urban extract, investigating the optimal values of plant extract to phospholipid ratio, reaction temperature and time. The optimal results were 3:1, 60 °C and 3 h, respectively, which yielded an entrapment efficiency of 95% *w*/*w*.

Jain et al. [165] developed rutin phytosomes with three different molar ratios and investigated their free radical scavenging activity via the DPPH radical scavenging assay. The study reported that the antioxidant activity increased concomitantly with the increase from a 1:1 to a 1:3 rutin to phospholipid ratio.

#### 5.2.5. Other Factors Affecting Phytosome Production

Several other factors can affect the production and yield of phytosomes, namely, production temperature and time, the use of cholesterol, ultrasound and agitation.

Matias et al. [166] produced *Plectranthus madagascariensis* (Pers.) Benth acetonic extract phytosomes and investigated the influence of the variation of three different parameters, i.e., the type of solvent (acetone, dichloromethane and ethanol), reaction time (1, 2 or 4 h) and the molar concentration of cholesterol (0, 2.5 and 5%). The optimal parameters, corresponding to particles with a mean size of 107.2 ±16.55 nm and a 93% %EE, were the use of acetone, a 2h reaction time and a concentration of cholesterol of 2.5%, although no statistical difference was found between the use of 2.5 and 5% cholesterol.

Saoji et al. [167] applied a QbD approach to optimize the production of standardized *Bacopa monnieri* (L.) Wettst. extract phytosomes regarding the molar ratio of extract to phospholipid, reaction temperature and time, with the optimal conditions found to be 3:1, 60 °C and 3 h, respectively, with an 87.09%EE. This formulation was then analyzed, and shown to have a 395 ± 11 nm mean particle size and −37.6 ± 1.1 mV. The in vitro drug release and antidepressant activity of the NPs in rats were also assessed. Over 11 h, 97% of the extract had been released from the phytosomes, compared to 42% in the pure extract. In vivo studies were performed in which rats were submitted to the Tail Suspension Test (TST) and Forced Swim Test (FST); in these tests, their immobility is measured and a decrease in the time of immobility is used to assess the efficacy of an antidepressant. The rats were treated with imipramine (10 mg/kg), pure extract (40 mg/kg) and the phytosomes (equivalent to 40 mg/kg). The decrease in immobility time were 44.8%, 23.4% and 46.9% for TST, respectively. For the FST, the immobility times were reduced by 43.9%, 22.8% and 45.6%, respectively.

Rasaie et al. [168] produced quercetin phytosomes with different molar ratios of cholesterol, evaluating their particle size and %EE. The lowest particle sizes were achieved with molar ratios of 1:2:0 and 1:2:0.2 (quercetin, phospholipid and cholesterol), i.e., 79 and 82 nm, respectively. They also evaluated the physical stability of the produced phytosomes. The phytosomes without cholesterol displayed a six-fold increase in size over a seven-day period, while the phytosomes with cholesterol displayed physical stability over a 21-day period, with little increase in size.

Nazari et al. [154] produced garlic essential oil phytosomes as a food preservative and evaluated three methods for size reduction: homogenization, probe sonication and a combination of the two. Both methods yielded particles with a size below 200 nm (161 ± 15 and 135 ± 17 nm, respectively). The combination of the methods produced even smaller particles (115 ± 21 nm) with lower turbidity, which is good for food applications. The encapsulation efficiencies obtained for the different methods were 91, 74 and 85%, respectively. The combination of methods also resulted in particles with higher stability after a 30-day storage period, with particle size increasing to approximately 200 nm, while for the homogenization method, this increased to approximately 400 nm. They also displayed a zeta potential of –12.36 ± 1.8 mV, and a polydispersity index of 0.34 ± 0.05, both being the lowest results of the three methods used. The antioxidant activity was also measured by DPPH scavenging activity, with the essential oil having a slightly higher antioxidant activity than the prepared nanophytosomes. The in vitro release was also assessed, with the essential oil releasing approximately 90%, while the nanophytosomes released only 66%. Therefore, nanophytosomes may lead to the creation of durable preservatives for food applications.

Jiao et al. [106] developed polypeptide-decorated nanoliposomes to improve the delivery of lutein. The developed formulations (free lutein, uncoated nanoliposomes and nanoliposomes coated with 0.04, 0.06 and 0.08%, *w*/*v* poly-L-lysine) were then analyzed for several parameters. The coated nanoliposomes displayed a particle size from 264 to 367 nm and a zeta potential from –38.6 to –27.9 mV. All formulations displayed encapsulation efficiencies above 90%. The authors also evaluated intestinal stability, observing a reduction in the degradation in SGF and SIF from 46.15 and 37.29% in free lutein to 30.95 and 27.67% in coated nanoliposomes; in vitro release, which increased from 43.28 to 51.26% in SGF and from 53.79 to 70.32% (uncoated and coated nanoliposomes); antioxidant activity, through DPPH, finding that scavenging activity increased from 10.46% in free lutein to 56.22% in coated nanoliposomes; and anticancer activity, observing that after 24 h, cell proliferation was 55.56% higher in cells exposed to uncoated nanoliposomes when compared to the coated nanoliposomes. 

One of the reasons for the increased attention recently given to phospholipid-based drug delivery systems is their use of naturally occurring phospholipid molecules. Their structural components, like the lipid contents of mammalian cell membranes, make them highly compatible with the human physiological system. Phytosomes can penetrate the lipoidal membrane of cells without the need for energy usage and in a noncytotoxic manner. The most used phospholipids are derived from soybean and have been shown to be free from any acute or chronic effects on laboratory animals, even at higher than recommended doses [118].

Phytosomes have gained increased attention lately, with a variety of research being carried out on their use as nanocarriers. In Table 3, some examples of the research that has been conducted and its procedures are summarized.

In some of these studies, the extract was characterized via HPLC prior and after phytosome formation in order to establish of the ability of the produced phytosomes to encapsulate the compounds present in the extract. Direito et al. [153] determined the concentration of nine phenolic compounds present in a persimmon extract. Gallic acid was the most abundant compound, with a concentration of 2.794 ± 0.263 mg/ 100 g fresh weight, followed by epicatechin, with a concentration of 0.401 ± 0.045 mg/ 100 g fresh weight. It was also concluded that the produced phytosomes were able to encapsulate 97.4% of the total phenolic compounds and 99.3% of the gallic acid present in the extract.

Mancini et al. [150] incorporated *Anonna muricata* L. into phytosomes and used HPLC-DAD to evaluate the efficiency in the incorporation of the compounds present in the plant extract. The initial analysis detected 22 compounds, namely epicatechin (6.23 ± 0.01 mg/ g extract, dry weight), quercetin-pentosyl-rhamnoside (5.33 ± 0.05 mg/ g extract, dry weight) and rutin (5.11 ± 0.01 mg/ g extract, dry weight). All 22 compounds were also found in the analysis of the phytosomes, which displayed and effective incorporation of the plant extract.

Lim et al. [169] detected several compounds in a *Moringa oleifera* Lam. aqueous extract, namely, chlorogenic acid, kaempferol, quercetin, rosmarinic acid, rutin and vicenin-2. Along with these target compounds, another 122 compounds were identified. Upon the analysis of the phytosomes, it was found that rutin and vicenin-2 had not been encapsulated, while the other compounds had. Quercetin (82.8%) and kaempferol (52.2%) appeared to have the highest affinity for the phytosomes.

### 5.3. Encapsulation by Nanostructured Lipid Carriers

NLCs are an upgrade of SLNs, the first-generation lipid nanocarriers. SLNs are colloidal drug carriers, in which drugs are carried inside a matrix of lipids that are solid at body temperature [173].

Emulsions and liposomes are greatly limited in their ability to stabilize chemically labile bioactive compounds, and are also characterized by a lack of controlled release. This is due to the low viscosity of the oils, that allows the bioactive substance to diffuse into the aqueous phase. This can be overcome by SLNs, where the solid matrix prevents the bioactive compound from being degraded [91].

SLNs have shown promising results against yeasts and dermatophytes due to their deep cellular penetration, longer retention times and higher concentrations [174]. Additionally, they use physiological lipids, do not require the use of organic solvents and can easily be produced on a large scale [175]. However, these nanocarriers present some disadvantages, namely, low loading capacity, because of their perfect crystalline structure, and the expulsion of the bioactive compounds due to the crystallization process which occurs during storage. Another drawback is the initial burst release which usually occurs with these formulations [176].

In order to overcome these disadvantages, a new lipid carrier was created. NLCs were developed by replacing a fraction of the solid lipids with liquid lipids to form the drug incorporating matrix [177]. The presence of the liquid lipids provides a better solubility of the bioactive ingredients, enhancing the loading efficiency while preserving the physical stability of the nanocarriers [178].

**Figure 6:**
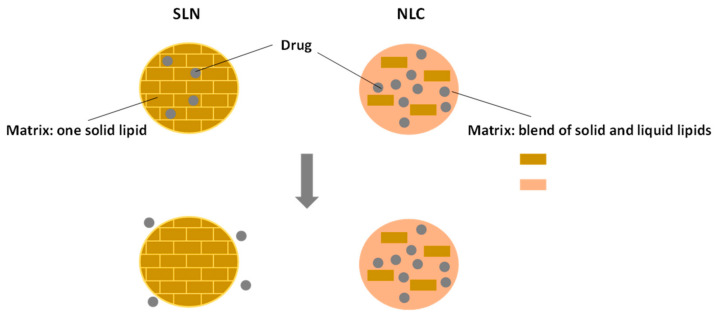
Structure of SLNs and NLCs. Adapted from [10].

SLNs and NLCs are very similar (Figure 6), both in their production processes and in the composition of their cores/matrices. In SLNs, solid lipids form a spatially stable composition, with very few imperfections being capable of entrapping the bioactive ingredient. With the addition of a liquid lipid, imperfections are expected to appear in the core of the nanocarrier, allowing for higher loading efficiency [175].

The main components involved in the preparation of SLNs and NLCs are solid lipids, liquid lipids (in the case on NLCs), surfactant, water and the compound to be incorporated. Other compounds, such as additives and microbial preservation agents, can also be added [179].

#### 5.3.1. Lipids

Lipids are the main structural material of lipid nanoparticles, up to 30% *w*/*w*, being responsible for the main component of the matrix, and being largely responsible for the properties of these colloidal systems, namely size, polydispersity, surface charge, short and long-term stability, drug loading and release profile. The main lipids used are free fatty acids, fatty alcohols, glycerol esters and waxes. Some of these lipids have surfactant properties that favor the formation of NPs [179,180].

Unlike NEs, in which the lipid phase is composed of oils, in SLNs and NLCs, a proportion of the oil is replaced by solid lipids, i.e., 100% in the case of SLNs and 70:30 to 99.9:0.1 in NLCs [181].

Yasir and Sara [182] tested the solubility of haloperidol in solid lipids by evaluating the amount of melted lipid required to dissolve 20 mg haloperidol. The tested lipids were glyceryl monostearate, Compritol 888 ATO, Precirol ATO 5, stearic acid and palmitic acid, and the amount required was 47.66 ± 0.95, 49.51 ± 0.83, 55.34 ± 2.24, 82.89 ± 2.10 and 142.37 ± 2.06 mg, respectively. Therefore, glyceryl monostearate was chosen as the solid lipid to produce haloperidol SLNs.

Hady et al. [183] screened the solubility of levofloxacin and doxycycline in five different lipids (stearic acid, Compritol 888 ATO, glyceryl monostearate, Gelucire 50/13 and Carnauba wax). The compounds displayed higher solubility in stearic acid (approximately 110 mg/g and 90 mg/g for levofloxacin and doxycycline, respectively), followed by Compritol 888 ATO (approximately 80 mg/g and 65 mg/g for levofloxacin and doxycycline, respectively). Both lipids were selected for the development of SLNs to increase the brain uptake of a levofloxacin–doxycycline mixture.

Devkar et al. [184] screened solid and liquid lipids to determine which were the best for the production NLCs for nose to brain delivery of ondansetron hydrochloride NLCs. The screened solid lipids were Compritol 888 ATO, glyceryl monostearate and Precirol ATO 5, while the liquid lipids tested were Capryol 90 and oleic acid. The solubilities obtained were 10 ± 2, 52.66 ± 3.05 and 24 ± 1 mg/g, and 10 ± 1 and 7.33 ± 1.52 mg/mL, respectively. The chosen lipids were glyceryl monostearate and Capryol 90.

Tamjidi et al. [185] screened different solid and liquid lipids for the development of astaxanthin NLCs. The liquid lipids displayed different solubilities of astaxanthin, i.e., from highest to lowest, oleic acid (1.96 mg/mL), olive oil (1.77 mg/mL), soybean oil (1.59 mg/mL) and corn oil (1.59 mg/mL). Afterwards, three different solid lipids were assessed, glyceryl behenate, glycerol monostearate and stearic acid. All three were miscible with the lipid liquid, but glyceryl behenate was chosen because the NLCs prepared with this lipid were stable, while the other two formulations yielded a white sediment after several hours. Afterwards, two variables were used optimize the formulation by response surface methodology: lipid phase to Tween 80 ratio, and oleic acid content of the lipid mixture. The optimal formulation was found to have these parameters: 1.8% and 22.4%, respectively. These resulted in NLCs with a mean particle size of 94.56 nm, a polydispersity index of 0.234, and a zeta potential of −24.37 mV.

Lacatusu et al. [186] utilized different concentrations of fish oil (10, 20 or 30%) and lutein (0.04, 0.08 or 0.12%) to optimize lutein NLCs. The optimized formulation (30% fish oil content and 0.08% lutein) displayed an average diameter of 167.5 nm, a polydispersity index of 0.172, a zeta potential of −34.2 mV and encapsulation efficiency of 88.5%. The in vitro antioxidant activity was evaluated, with the results showing the ability to scavenge up to 98% of oxygen free radicals. Lastly, an in vitro drug release assessment displayed a better sustained release when compared to conventional NEs.

#### 5.3.2. Surfactants

Surfactants, and sometimes cosurfactants, are used in the production of SLNs and NLCs to diminish the interfacial tension between the lipid and the aqueous phase, and to prevent aggregation. It has been reported that these substances influence the crystalline structure of the particles and determine their electrokinetic behavior [179,187].

Martins et al. [188] produced camptothecin SLNs using three different lipids (cetyl palmitate, Dynasan 14 and Witepsol E85) and four different surfactants (polysorbate 20, 40, 60 and 80) at different concentrations (5% and 15% for lipids, 0.8% and 2.0% for surfactant), for a total of 27 formulations; they then studied their characteristics on the day of production and after one year of storage. The authors, based on the SLN characteristics (i.e., a mean size ≤ 200 nm, homogenous size distribution, low number of microparticles, good storage stability and EE above 90%), considered seven formulations as most suitable. The findings confirmed that the most suitable formulations were composed of cetyl palmitate as the lipid (in six out of seven formulations), and polysorbate 20, 60 or 80 as the surfactant (for one, three and three of the formulations, respectively).

Salem et al. [189] attempted to optimize an almotriptan maleate NLC formulation by a D-optimal design with four variables: ratio of solid to liquid lipid (50:50, 70:30 or 90:10), type of solid lipid (Compritol 888 ATO, Precirol ATO 5 and stearic acid), type of cosurfactant (Labrasol, Lauroglycol 90 and Transcutol HP) and the effect of chitosan coating. A total of 13 formulations were produced; the formulation with a 70:30 ratio, Compritol 888 ATO as solid lipid and Lauroglycol 90 as cosurfactant (2:1 ratio polysorbate 80:Lauroglycol 90) displayed the lowest particle size and highest %EE, 285.61 ± 3.32 nm and 79.41 ± 0.14%, respectively. Using the Design expert software, an optimal formula was found, consisting of Compritol as solid lipid in a 50:50 ratio and lauroglycol as the cosurfactant, choated with chitosan. This formula displayed a particle size of 254.93 ± 1.85 nm and 80 ± 0.32%EE.

Shofia et al. [71] encapsulated brown seaweed (*Sargassum longifolia)* polysaccharides in orange oil NEs and NLCs. The developed NPs had an average diameter of 170 and 153 nm, respectively. They also displayed zeta potential values of −43.9 and −60 mV and encapsulation efficiencies of 67.29% and 78.7%, respectively. An in vitro drug release analysis showed a slow and sustained release with both types of NPs, with 80% being released from NEs and 95% from NLCs after 12 h. Table 4 and Table 5 summarize several published studies where SLNs and NLCs were produced to incorporate neuroprotective compounds.

## 6. Comparison of Lipid-Based Nanoparticles with Other Types of Nanoparticles for Brain-Delivery

Lipid-based, polymeric and inorganic nanoparticles have been extensively studied for the effective delivery of drugs across the BBB via a noninvasive pathway [170].

The polymers used in NP formulation can be natural or synthetic. Some of the most widely used natural polymers are chitosan, cellulose and gelatin. As for synthetic polymers, polylactic acid and poly-(lactide-coglycolide) are commonly used. These NPs have been widely researched due to their potentially high versatility and biocompatibility, depending on the chosen polymer [170,203,204].

The use of polymer NPs has a few critical disadvantages which has hindered their market application, such as the toxicity of some of the polymers, the presence of solvent residues during production and purification, high cost, degradability, difficulty in the application of large-scale production and the requirement for high quality and high purity polymers [204].

Inorganic NPs display some advantages over polymeric NPs, namely, in terms of the control of size and shape, the ease of production and functionalization and ease of tracking by microscopy or analytic techniques. They also provide a stronger support for the nanostructure than organic-based structures. These NPs are made using heavy metals or semiconductive metals such as gold, silica or carbon. However, these NPs have some disadvantages, e.g., they might not be degraded or eliminated through the kidneys, or may cause undesired long-term toxicity [170,205].

Lipid-based NPs have several advantages that justify increased investment in their development. These types of particles display protection against chemical and enzymatic degradation, lipid compatibility, gradual release of active compound from the lipid matrix, diminished adverse side effects and chronic toxicity. Furthermore, they hold the possibility of production without the use of organic solvents (e.g., via the high-pressure homogenization method) and easy scale-up [203,204].

Of the many NPs being investigated by scientists, lipid-based NPs have taken the lead, due to the aforementioned advantages, along with their biocompatibility and versatility. These NPs can be formulated in different ways to meet a wide range of product requirements, namely, disease condition, administration route, as well as other parameters, such as cost, stability, toxicity and efficacy. Their proven safety and efficacy have made them attractive candidates for the formulation of pharmaceuticals [206].

Therefore, lipid-based NPs are considered to be at the forefront of the quickly evolving field of nanotechnology, showing great potential for use in drug delivery and clinical medicine [206].

A lot of formulations have displayed the efficacy of nanocarriers in transporting therapeutic molecules across the BBB at the cellular and animal levels. Despite this, few have been approved for clinical uses, while for other diseases (i.e., cancers and cardiovascular diseases), these have already been marketed or clinically approved [170].

One of the difficulties associated with the study of crossing the BBB is the models utilized. Differences may arise between the brain microenvironments of different species, hampering the ability to predict the behavior in humans of formulations tested on other animals. The appearance of innovative models, such as organs-on-chips, may help overcome this [170].

## 7. Conclusions

Lipid-based nanoparticles have shown tremendous potential in the prevention and treatment of neurological diseases, which are having a significant impact on the lives of an growing number of people.

One of the biggest obstacles to the treatment of these diseases is the ability to deliver drugs directly to the brain, mainly due to the tightness of the BBB to most drugs. Nanoencapsulation appears to be a possible way to circumvent this obstacle.

Lipid-based NPs show some advantages compared to polymeric and inorganic NPs. This has propelled them to the forefront of investigation, with a vast amount of research being done to optimize formulations that will allow for higher brain uptake.

In this review, different types of lipid-based NPs were discussed, along with the critical parameters in their formulation. Furthermore, a summary of the existing literature on each type of NP was presented, with the formulation parameters being given and main results discussed.

Moving forward, further research is necessary in this field, in terms of both optimizing the formulations to improve the bioavailability of active compounds and brain uptake. The development of models closely resembling the in vivo human brain environment may be a critical step for the optimization of said particles. This may, in turn, give rise to the creation of clinically approved therapeutics.

## Figures and Tables

**Table 1:** Nanoemulsions produced to encapsulate neuroprotective compounds and extracts.

Compounds/Extracts	Oil	Surfactant: Cosurfactant	Oil: Surfactant Ratio *v*/*v*	T	Oil Phase	Aqueous Phase	Ultrasound	Rotation	Time	Analysis	Ref
**Seaweed extract**											
*Sargassum longifolium* (Turner) C.Agardh	Orange oil	Span 80: Pluronic L81	10:1	Room T	Oil, Span 80	Deionized water, Pluronic L81	Probe sonicator, 30 s on; 10 s off; 40% amp, 140 W, 30 min	Yes, 30 min	24 h	DLS, SEM, TEM, FTIR, UV-Vis, in vitro drug release, cytotoxicity	[71]
**Natural compounds**											
Astaxanthin	Soybean oil	Ginseng saponins				0.08–1.2% *w*/*w* surfactant, distilled water		8000 rpm	5 min	DLS, UV-Vis	[67]
Astaxanthin	Soybean oil	Modified lecithin or sodium caseinate			Oil, drug	Distilled water, 2.0% *w*/*w* surfactant		10,000 rpm	5 min	DLS, laser diffraction, UV-Vis, in vitro intestinal digestion, CLSM	[68]
Carvacrol	*n*-hexane	Tween 80			Oil, drug	Deionized water, surfactant	20 kHz, 100 W, 1 h	1 h	2 h	TEM, DLS, *in* *vivo* studies, ELISA, HPLC	[72]
Kaempferol	16% *w*/*w* MCT	1% *w*/*w* Polysorbate 80			Oil, 0.1% drug, 5% *w*/*w* lecithin	Distilled water, surfactant		9500 rpm	1 min	PCS, TEM, viscometry, refractometry, HPLC, DPPH, ex vivo diffusion, in vivo studies	[73]
Lutein	10% corn oil (*w*/*w*)			50 °C	Oil, ethyl acetate, drug	Distilled water, 2% whey protein isolate			20 min	DLS, TEM, HPLC, in vitro cytotoxicity, cellular uptake	[60]
Lutein	10 g soybean oil, 0.67 g Xangold 15% oil, 12 drops vitamin E oil	8 g Phospholipon 85G		60 °C	Oils, surfactant, drug	Natural spring water			35 min	DLS, HPLC-DAD	[61]
Lutein	Corn oil	Quillaja saponin, Tween 80, WPI or sodium caseinate			Oil, drug	0.25% surfactant, aqueous buffer solution, 10 mM phosphate, pH 7		10,000 rpm	2 min	Colorimeter, DLS	[69]
Lutein	EtOh/ MCT oil 50/50, *v*/*v*	Lecithin, β-lactoglobulin, Biozate 1 or Tween 20			Oil, drug, antioxidant, lecithin	β-lactoglobulin, Biozate 1 or Tween 20 in water	5 min 70% max power	500 rpm	10 min	Laser diffraction, UV-Vis, HPLC, cytotoxicity, cellular uptake	[70]
Lycopene	Sesame, linseed, walnut oil				Oil, drug	Deionized water, lactoferrin		10,000 rpm	3 min	DLS, TEM, UV-Vis, in vitro digestion	[74]
Naringenin	Capryol 90	Tween 20: EtOH			Oil, drug, surfactant	Distilled water		Continuous	> 1 h	PCS, TEM, ROS activity, in vitro studies	[75]
Rutin	Sefsol 218: Tocopheryl polyethylene glycol 1000 succinate	Solutol HS15: Transcutol P	1:9; 1:8; 1:7; 1:6; 1:5; 1:4; 1:3.5; 1:3; 3:7; 1:2; 4:6; 5:5; 6:4; 7:3; 8:2; 9:1		Oil, drug, surfactant	Double distilled water		Continuous		TEM, HPLC, DLS, in vitro drug release, in vivo studies	[76]
siRNA	Flaxseed oil	Tween 80			DOTAP dissolved in EtOH, siRNA, oil	Water, Lipoid E80^®^, surfactant	10 min 22% amplitude 50% duty cycle	6000 rpm	2 min	DLS, TEM, EMSA, CLSM, FCM, in vitro gene silencing, in vivo studies	[77]
Thymoquinone	Oleic acid	Tween 20: carbitol (0:3; 1:3; 2:3; 3:3; 3:2; 3:1; 3:0)	1:9; 2:8; 3:7; 4:6; 5:5; 6:4; 7:3; 8:2; 9:1		Oil, drug, surfactants	Purified water		Continuous		DLS, DPPH, FTIR, refractometry, in vitro drug release, ex vivo permeability, in vivo studies, UPLC-PDA	[78]
**Synthetic compounds**											
Asenapine maleate	Capmul PG-8	Kolliphore RH40:Transcutol HP (3:1; 2:1; 1:1; 1:2; 1:3)	1:9; 2:8; 3:7; 4:6; 5:5; 6:4; 7:3; 8:2; 9:1		Oil, drug, surfactant	Double distilled water		Continuous		DLS, TEM, viscometry, in vitro drug release, in vivo studies, HPLC	[79]
Darunavir	Soybean oil	Tween 80 (0.5; 0.75; 1; 1.25; 1.5% *w*/*v*)		55–60 °C	8% *w*/*v* oil, surfactant, drug	Distilled water, 1.2% egg lecithin		8000 rpm	20 min	DLS, UV-Vis, conductivity, TEM, in vitro drug release, in vivo studies	[80]
Gabapentin	Capmul MCM	Tween 80: PEG-400	1:9; 2:8; 3:7; 4:6; 5:5; 6:4		Oil, surfactants, drug	Double distilled water, 0.3% oleic acid, 2.25% glycerol	70% amplitude		10 min	UV-Vis, FTIR, DLS, TEM, viscometry, in vitro diffusion, *ex* *vivo* permeation	[81]
Indinavir	10% *w*/*v* soybean oil	0.2, 0.6, 1% *w*/*v* Tween 80		70 °C	Oil, surfactant, 0.25% *w*/*v* α-tocopherol, 1.2% *w*/*v* egg PC		750 W, 20 min at 50% amplitude	15,000 rpm	3 min	DLS, in vitro drug release, HPLC, FLM, in vivo studies	[82]
Letrozole	Triacetin	Tween 80:PEG-400 1:1; 2:1; 3:1; 4:1	1:9; 1:8; 1:7; 1:6; 1:5; 2:8; 1:3.5; 1:3; 3:7; 1:2; 4:6; 5:5; 6:4; 7:3; 8:2; 9:1		Oil, drug, surfactant	14.66% cosurfactant, 48% distilled water		Yes		UV-Vis, DLS, TEM, in vitro drug release, ex vivo permeation, DSC, in vivo studies	[83]
Risperidone	MCT: soybean oil (4:1 *w*/*w*)	2% *w*/*w* Polysorbate 80, Kolliphor^®^ P 188 or Solutol^®^ HS 15		25 or 50 °C	20% Oil, 2% emulsifier (lecithin), 0.05% antioxidant (BHT). 2% benzyl alcohol	Surfactants dissolved in 0.01M PBS pH 9 or double distilled water		10,000 rpm	3 min	PCS, laser diffraction, viscometry, conductivity, AFM, DSC, FTIR, in vivo studies	[64]
Risperidone (follow-up study)	MCT: soybean oil (4:1 *w*/*w*)	2% *w*/*w* Polysorbate 80		50 °C	20% Oil, 2% emulsifier (lecithin), 0.05% antioxidant (BHT). 2% benzyl alcohol	Double-distilled water, Polysorbate 80, sodium oleate, glycerol		10,000 rpm	3 min	PCS, laser diffraction, DLS, viscometry, conductivity, in vivo studies	[84]
Risperidone	Capmul MCM	Tween 80: (transcutol: propylene glycol (1:1 *w*/*w*))		35 °C	8% oil, 29.33% surfactant, drug	0.5, 0.7% Carbopol 934		Continuous		UV-Vis, PCS, in vivo studies	[85]
Rivastigmine hydrochloride	Capmul MCM	Tween 80:Transcutol P (1:1; 1:2; 2:1; 3:1; 4:1; 5:1)	1:9; 1:8; 1:7; 1:6; 1:5; 2:8; 1:3.5; 1:3; 3:7; 1:2; 4:6; 5:5; 6:4; 7:3; 8:2; 9:1		Oil, drug, surfactant	Distilled water		Continuous		HPLC, UV-Vis, DLS, TEM, in vitro drug release, in vivo studies	[66]
Saquinavir	Flaxseed or safflower oil	Egg PC: deoxycholic acid		60 °C	Oil, drug, ethanol		10 min, 21% amplitude	Continuous		DLS, TEM, in vivo studies	[86]
Tetrabenazine	Capmul MCM	Tween 80: Transcutol P 1:0; 1:1; 1:2; 1:3; 2:1; 3:1; 4:1 *v*/*v*	1:9; 1:8; 1:7; 1:6; 1:5; 1:4; 3:7; 1:2; 4:6; 5:5; 6:4; 7:3; 8:2; 9:1	25 °C	Oil, drug, surfactant	HPLC-grade water		High speed		HPLC, UV-Vis, DLS, refractometry, ex vivo nasal permeation, in vivo studies	[63]
Topiramate	Capmul MCM C8	2:1 Tween 20:Carbitol			Oil, surfactant, drug	Water		100 rpm	30 min	DLS, TEM, in vivo studies	[87]
Valproic acid	MCT: safflower seed oil (1:3 *w*/*w*)	Tween 80		60 °C	Oil, drug, 1% *w*/*w* lecithin		24 kHz, 240 W	Yes	15 min	DLS, TEM	[88]

Abbreviations: AFM—Atomic Force Microscopy; BHT—Butylhydroxytoluene; CLSM—Confocal Laser Scanning Microscopy; DLS—Dynamic Light Scattering; DOTAP— (N-[1-(2, 3-dioleoyloxy) propyl]-N, N, N-trimethylammoniumsalt); DPPH—2,2-diphenyl-1-picrylhydrazyl; DSC—Differential Scanning Calorimetry; ELISA—Enzyme-Linked Immunosorbent Assay; EMSA—Electrophoretic Mobility Shift Assay; EtOH—Ethanol; FCM—Flow Cytometry; FLM—Fluorescence Microscopy; FTIR—Fourier-transform Infrared Spectroscopy; HPLC—High-Performance Liquid Chromatography; MCT—Medium-chain Triglycerides; PC—Phosphatidylcholine; PEG-400—Polyethylene glycol 400; PCS—Photon Correlation Spectroscopy; ROS—Reactive Oxygen Species; siRNA—Small interfering RNA; T—Temperature; TEM—Transmission Electron Microscopy; UPLC-PDA—Ultra Performance Liquid Chromatography coupled with Photodiode Array Detector; UV-Vis—Ultraviolet-visible spectroscopy; WPI—Whey Protein Isolate.

**Table 2:** Nanoliposomes produced to encapsulate neuroprotective compounds

Compound	PP	Solvent	Molar Ratio (E: PP)	T	Chol	Ultrasound	Rotation	Time	Rehydration Film	Analysis	Ref
**Natural compounds**											
7,8-dihydroxy-flavone	Soybean PC	Absolute EtOH	1:40, 1:30, 1:15, 1:10, 1:5 *w*/*w*	25 °C	5:1 PC:Chol	40 kHz, 5 min, 10 s on/off	Yes	30 min	PBS pH 7.4	DLS, UPLC, TEM, FTIR, DSC, DPPH, in vitro drug release	[96]
β-carotene	Marine PP or egg PC	5 mL absolute EtOH	0.01, 0.02, 0.03, 0.04 g to 1g PP	55 °C		9 min 240 W 8 s on/off	Yes	60 min	100 mL PBS pH 7.4	DLS, TEM, DSC, UV-Vis	[97]
Baicalein	DPPC	Chloroform for DPPC, MetOH for baicalein	1:3 1:5 1:10	37 °C	15% Chol, 10% PEG2000 PE	30 s	150 rpm	2 h	PBS pH 7.4	TEM, DLS, UV-Vis, HPLC, DSC, DPPH, CLSM, cellular uptake	[98]
Basic fibroblast growth factor	Hydrogenated soy PC	1 mL Poloxamer 188-grafted heparin copolymer 5% *w*/*v*		5 °C		110 W, 20 s	2500 rpm	5 h	Double distilled water	TEM, DLS, viscometry, ELISA, UV-Vis, in vivo studies	[99]
Curcumin	Soy, rapeseed, salmon lecithin	48.5 mL distilled water	10 mg curcumin to 1.5 g lecithin			40 kHz 40% full power, 120 s, 1 s on/off	Yes	5 h		DLS, HPLC, TEM, in vitro anticancer activity evaluation	[100]
Curcumin	DPPC	Chloroform: MetOH (2:1 *v*/*v*)	1:5	45 °C	2:1 PP:Chol	Yes			PBS pH 7.4	DLS, FCM, in vivo studies, brain cell studies	[101]
Curcumin	DSPC	Chloroform: MetOH (2:1 *v*/*v*)	(0.375–0.75):2:1 E:PP:Chol	60 °C	Yes	Yes	Yes	1 h	PBS pH7.4 or FITC-dextran	DLS, *post* *mortem* brain tests, cellular uptake	[102]
Cytarabine	Lipoid E80	EtOH		Room T	Yes		700 rpm	15 min		PCS, HPLC, TEM, in vitro drug release, stability and cell uptake, CLSM	[103]
Docosa-hexaenoic, eicosa-pentaenoic acid (2:3) *w*/*w*	Soybean PP	Deionized water and glycerol (2% *v*/*v*)	0.4:2	30 °C		20 kHz 120 W (10, 15, 20 min)	600, 800, 1000 rpm	30, 45, 60 min		DLS, GC	[104]
Galantamine	DSPC, DSPE	Chloroform: MetOH (9:1 *v*/*v*)		65 °C	Yes	Probe sonicator	60 rpm	30 s	PBS pH 7.4 or 5% *w*/*v* dextrose	FTIR, UV-Vis, DSC, DLS, TEM, in vitro drug release	[105]
Lutein	Lecithin	Absolute ethanol	1:10:40:10 lutein, chol, Tween 80, lecithin	50 °C	Yes			30 min		HPLC, DLS, TEM, FTIR, in vitro drug release, antioxidant activity	[106]
OX-26 or IgG	PC, DSPC			37 °C for PC, 53 °C for DSPC	20:10:0.8: (0.002–0.02) DSPC:Chol:DSPE-PEG_2000_:DSPE-PEG_2000_-Biotin	Probe sonication		1 h	Excess solution of biotin-OX-26 or biotin-IgG	ELISA, DLS, cellular uptake, TEM, CLSM	[107]
Quercetin, rosmarinic acid	PA, DPPC, DHDP	MeOH for quercetin, chloroform for PP, PBS for RA		25 °C	5:4:1 DPPC:Chol:DHDP	46 kHz		50 min	1 mL aqueous solution containing RA	HPLC, ELISA, DLS, SEM, TEM, XPS, in vivo studies	[108]
**Synthetic compounds**											
Beclometha-sone dipropionate	Lipoid E80	EtOH		Room T	Yes		700 rpm	15 min		PCS, HPLC, TEM, in vitro drug release, stability and cell uptake, CLSM	[103]
Cisplatin	Soy PC	Silver nitrate for cisplatin, chloroform: diethyl ether (3:1 *v*/*v*) for PP	(9:1:1.5 mg) PC, PE-PEG, QCS-modified PP	25 °C			Yes		5 mL PBS pH 7.4 and aqueous cisplatin (100 μg/ 1 mg lipid)	AAS, AFM, DLS, in vitro drug release, cellular uptake	[109]
Flucytosine	Soybean PC	Chloroform: MetOH 3:2 *v*/*v*			1:1:1:(0, 0.25, 0.5 or 0.75) PC: Chol: Span 65: Glutathione	Ultrasonic bath 30 min	90 rpm	1 h	15 mL aqueous solution of flucytosine in PBS pH 7.4	UV-VIS, DLS, TEM, in vitro drug release, cellular uptake, in vivo studies	[110]
Lamotrigine	Lipoid 90G	Chloroform: MetOH (2:1 *v*/*v*)	Various	35 °C	Yes	Yes	Yes	1 h	Tween 80, nasal saline buffer pH 6.5	PCS, UV-Vis, TEM, DSC, XRD, in vitro drug release, CLSM	[111]
Metmorfin Hydrochloride	PS	Pure EtOH		50 °C		2 min mild frequency	800 rpm		Distilled water	DLS, TEM, UV-Vis, in vivo studies	[112]
Phosphatidic acid, cardiolipin	Bovine brain sphingomyelin	Chloroform: MetOH (2:1 *v*/*v*)	5% either extract	55 °C	1:1 molar ratio Chol:PP mixed with 2.5% mal-PEG-PE			3 h	PBS	DLS, CLSM, cellular uptake	[113]
Quetiapin fumarate	Egg PC	10 mL MetOH: chloroform 2:1	1:1; 1:2; 1:3	37 ºC	1:1; 1:2; 1:3 Chol:PC	2 min 80% amplitude	90 rpm	2 h	10 mL nasal saline buffer pH 6.8	FTIR, SEM, TEM, DLS, ex vivo drug diffusion, in vitro drug release, in vivo studies	[114]
Tempamine	Egg PC or HSPC	Tert-butanol for lipids, 70% EtOH for drug	54:41:5 PP:Chol: ^2000^PEG-DSPE	60 ºC for HPSC, room T for egg PC					250 mM ammonium sulfate	EPR, in vivo studies	[115]
Teriflunomide	Lipoid S100	10 mL chloroform: MetOH(95:5 *v*/*v*)	5, 7.5 or 10 mg drug to 75, 100 or 125 mg PP		Yes	70% amplitude 2–5 min	Yes		PBS pH 7.4	DLS, TEM, SEM, DSC, PXRD, UV-Vis, in vitro drug release, in vivo studies	[116]

Abbreviations: 2000PEG-DSPE—N-carbamyl-poly-(ethylene glycol methyl ether)-1,2-distearoyl-sn-glycero-3-phosphoethanolamine triethyl ammonium salt; AAS—Atomic Absorption Spectroscopy; AFM—Atomic Force Microscopy; Chol—Cholesterol; CLSM—Confocal Laser Scanning Microscopy; DHDP—Dihexadecyl Phosphate; DLS—Dynamic Light Scattering; DPPC—Dipalmitoylphosphatidylcholine; DPPH—2,2-diphenyl-1-picrylhydrazyl; DSC—Differential Scanning Calorimetry; DSPC: 1,2-distearoyl-sn-glycerol-3-phosphatidylcholine; DSPE-PEG2000—1,2-distearoyl-sn-glycerol-3-phosphoethanolamine-N-[methoxy(polyethyleneglycol)-2000]; DSPE-PEG2000-Biotin—1,2-distearoyl-sn-glycerol-3-phosphoethanolamineN-[biotinyl (polyethyleneglycol)-2000]; E—Extract; ELISA—Enzyme-Linked Immunosorbent Assay; EPR—Electron Paramagnetic Resonance; EtOH—Ethanol; FCM—Flow cytometry; FITC—Fluorescein Isothiocyanate; FTIR —Fourier-transform Infrared Spectroscopy; GC—Gas Chromatography; HPLC—High-Performance Liquid Chromatography; HSPC—Hydrogenated Soy Phosphatidylcholine; IgG—Immunoglobulin G; OX-26—Anti-Transferrin Monoclonal Antibody; mal-PEG-PE—1,2-stearoyl-sn-glycero-3-phosphoethanolamine-N- [maleimide(poly(ethylene glycol)-2000)]; MetOH—Methanol; PA—Phosphatidic Acid; PBS—Phosphate Buffer Solution; PC—Phosphatidylcholine; PCS—Photon Correlation Spectroscopy; PE-PEG—1,2-distearoyl-sn-glycero-3-phosphoethanolamine-N-[amino(polyethylene glycol)-2000]; PEG2000 PE—1,2-distearoyl-sn-glycero-3-phosphoethanolamine-N- [methoxy(polyethylene glycol)-2000]; PP—Phospholipid; PXRD – Powder X-ray Diffraction; RA—Rosmarinic Acid; SEM—Scanning Electron Microscopy; T—Temperature; TEM—Transmission Electron Microscopy; UPLC—Ultra Performance Liquid Chromatography; UV-Vis—Ultraviolet-visible spectroscopy; XPS—X-ray Photoelectron Spectroscopy; XRD—X-ray Diffraction.

**Table 3:** Nanophytosomes intended to encapsulate neuroprotective compounds and extracts

Extracts/Compounds	PP	Solvent	Molar Ratio (E: PP)	T	Chol	Ultrasound	Rotation	Time	Rehydration Film	Analysis	References
**Plant extracts**											
*Annona muricata* L. aqueous extract	PC	3 mL THF	1:4	75 °C	32.5%	15 min	Yes	4 h	PBS	DLS, HPLC-DAD, FLS, MAO-A inhibition	[150]
*Moringa oleifera* Lam. aqueous extract	Soy lecithin	10 mL DCM	Various	Room T	Yes	60% amplitude for 15 min, 15 s on/30 s off	Yes	3 h	10 mL *n*-hexane	DLS, LC-MS, TEM	[169]
Persimmon	PC	20 mL EtOH	1:1; 1:2	25 °C			300 rpm	2 h	40 mL 2% acetic acid solution	HPLC, DPPH, DLS, Folin-ciocalteu, UV-Vis, UHPLC-DAD, in vivo studies	[153]
*Plectranthus madagasca-riensis* (Pers.) Benth.acetonic extract	PC	20 mL acetone, DCM or EtOH	1:1	50 °C	0, 2.5 or 5%		Yes	1, 2 or 4 h	40 mL reverse osmosis water	HPLC-DAD, SEM, DLS	[166]
Standardized *Bacopa monnieri* (L.) Wettst. extract	Phospholipon ^®^90H	40 mL EtOH	1:0.5; 1:1; 1:1.75; 1:2.5; 1:3	40, 44, 50, 56, 60 °C			Continuous at rehydration	1, 1.4, 2, 2.6, 3 h	*n*-hexane	HPLC, Phm, SEM, DLS, FTIR, DSC, TGA, PXRD, in vitro drug release, ex vivo permeation, in vivo studies	[167]
Standardized *Centella asiatica* extract	Phospholipon ^®^90H	40 mL EtOH	1:0.5; 1:1.01; 1:1.75; 1:2.49; 1:3	40, 44, 50 56, 60 °C			Continuous at rehydration	1; 1,4; 2; 2,6; 3 h	*n*-hexane	HPLC, Phm, SEM, PCS, FTIR, DSC, PXRD, in vitro drug release, in vivo studies	[152]
**Natural compounds**											
Apigenin	Phospholipon 90H (Hydrogenated soy PC)	1,4-dioxane: MetOH (14:6)	1:1; 1:2; 1:3	40, 50, 60 °C				2 h	100 mL *n*-hexane	UV-Vis, DLS, DSC, FTIR, H-NMR, PXRD, in vitro drug release, in vivo studies	[149]
Catechin	PC	DCM	1:1					3 h	30 mL *n*-hexane	UV-Vis, SEM, FTIR, DSC, PXRD, H-NMR, DPPH	[148]
Celastrol	Soy PC	Anhydrous EtOH	1:1; 1:2; 1:3	40 °C		Brief (2 min)	100 rpm	3 h	6 mL deionized water	UV-Vis, FTIR, DSC, PXRD, DLS, TEM, in vitro drug release, in vivo studies	[138]
Chrysin	Soy or egg PC	12.5 mL THF	1:2; 1:3	40 °C			Yes	4 h	12 mL distilled water	DLS, HPLC, AFM, FTIR, XRD, SEM, in vitro drug release	[170]
Curcumin	Hydrogenated soy PC	20 mL DCM	1:1	≤60 °C				2 h	10 mL *n*-hexane	HPLC, DSC, HPTLC, in vivo studies	[159]
Curcumin	PC	30 mL DCM	1:1; 1:2; 1:4	40 °C			Yes	2 h	50 mL *n*-hexane	SEM, TEM, HPLC, DSC, H-NMR, FTIR, PCS, in vivo studies	[160]
Embelin	Phospholipon ^®^90H	250 mL EtOH	1:0.5; 1:1; 1:2; 1:3	≤60 °C				2 h		UHPLC, UV-Vis, DSC, FTIR, PXRD, H-NMR, in vitro drug release	[147]
Hesperidin	Soy lecithin	40 mL DCM	1:0.5; 1:1; 1:2; 1:3	≤60 °C				2 h		UV-Vis, DSC, SEM, in vitro drug release	[164]
Quercetin	PC	MetOH: Chloroform (1:1 *v*/*v*)	1:2	45 °C	2:0.2 PC:Chol	Probe sonicator for 5 min	80 rpm		Glucose 50% solution	TEM, DLS, UV-Vis, DPPH	[151]
Rutin	PC	Absolute EtOH	1:1; 1:2; 1:3	45 °C				30 min	5 mL distilled water	DLS, SEM, UV-Vis, FRAP, FTIR, HPLC	[156]
Rutin	Soy PC	MetOH: Chloroform (1:4)	1:1; 1:2; 1:4	45 °C	Yes				Distilled water	DLS, DSC, FTIR, UV-Vis	[158]
Rutin	PC	20 mL DCM	1:1	45–50°C					100 mL *n*-hexane	HPLC, FTIR, DSC, PXRD, SEM, DPPH, in vitro drug release	[171]
Silymarin	Soy and egg yolk lecithin	100 mL MetOH	1:0.25; 1:0.5; 1:1; 1:2	Room T			Yes		300 mL petroleum ether	SEM, TEM, H-NMR, DSC, FTIR, HPLC, in vivo studies	[155]
Silymarin	Soy PC	20 mL absolute EtOH	1:5; 1:10; 1:15	25 °C		4min, 5 s on/off, 60% amplitude	180 rpm	2 h	PBS, pH 7.4	UV-Vis, FTIR, DSC, TEM, DLS	[172]

Abbreviatures: AFM—Atomic Force Microscopy; Chol—Cholesterol; DCM—Dichloromethane; DLS—Dynamic Light Scattering; DSC—Differential Scanning Calorimetry; DPPH—2,2-diphenyl-1-picrylhydrazyl; DSPE-PEG-maleimide—1,2-stearoyl-sn-glycero-3-phosphoethanolamine-N-[maleimide(-poly(ethylene glycol)-2000)]; E—Extract; EtOH—Ethanol; FLS—Fluorescence Spectroscopy; FRAP—Ferric Reducing Antioxidant Power; FTIR—Fourier-transform Infrared Spectroscopy; H-NMR—Proton Nuclear Magnetic Resonance; HPLC —High-Performance Liquid Chromatography; HPLC-DAD—High-Performance Liquid Chromatography coupled to Diode Array Detection; HPTLC—High-Performance Thin Layer Chromatography; LC-MS—Liquid Cromatography-Mass Spectrometry; MAO-A—Monoamine oxidase A; PBS—Phosphate Buffer Solution; PC—Phosphatidylcholine; Phm—Photomicroscopy; PP—Phospholipid; PXRD—Powder X-ray Diffraction; SEM—Scanning Electron Microscopy; T—Temperature; TGA—Thermogravimetric Analysis; THF—Tetrahydrofuran; TEM—Transmission Electron Microscopy; UHPLC—Ultra-High-Performance Liquid Chromatography; UHPLC-DAD—Ultra-High-Performance Liquid Chromatography coupled to Diode Array Detection; UV-Vis—Ultraviolet-visible spectroscopy; XRD—X-ray Diffraction.

**Table 4:** SLNs produced to encapsulate neuroprotective compounds.

Compound	Lipids	Solvent	T	Oil Phase	Aqueous Phase	Ultrasound	Rotation	Time	Analysis	Ref
**Natural compounds**										
Andrographolide	Compritol 888 ATO	5 mL acetone for lipids	50 °C oil phase, 75 °C aqueous phase	Drug, lipids, fluorescein isothiocyanate	30 mL water, Brij 78		Continuous		DLS, TEM, DSC, HPLC-DAD, HPLC-FLD, in vitro drug release, in vivostudies	[45]
BACE1 siRNA	200 mg Witepsol E 85 solid triglycerides	2 mL DCM for lipids	Room T	Drug, lipids, RVG-9R (to increase intracellular pathway), 10 mL polyvinyl alcohol (2% *w*/*v*)	Chitosan (1% *w*/*v*), water containing 1% *v*/*v* acetic acid and PVA (2% *w*/*v*)	30 s 70% amplitude	Yes		DLS, SEM, FMPR	[190]
Camptothecin	5 or 15% Cetyl palmitate, Dynasan 114 or Witepsol E85		5–10 °C above lipid melting point	Drug, lipid	Water, 0.8 or 2% surfactant (Polysorbate 20, 40, 60 and 80)		Yes		PCS, DSC, HPLC, in vitro drug release, in vivo studies	[188]
Curcumin	Compritol 888 ATO (7.27%)		82–85 °C	Lipid	Water, polysorbate 80 (45.45%), soy lecithin (0.58%), drug		5000 rpm	1.5h	CLSM, in vivo studies	[46]
Lutein	Fish oil, corn oil		85 °C	Fish oil, glycerol stearate, carnauba wax, corn oil, drug	Water, 4% surfactant mixture of Tween 80/ lecithin/ block copolymer		25,000 rpm	10 min	DLS, TEM, UV-Vis, DSC, antioxidant activity, in vitro drug release	[186]
Noscapine	Stearic acid (0.70 mM), egg PC (0.14 mM)		70 °C	Drug, lipids, sodium glycocholate (0.69 mM)	Distilled water (111.10 mM)		Yes		DLS, TEM, AFM, UV-Vis, FTIR, DSC, PXRD, in vitro drug release, in vivo studies	[191]
Resveratrol	Compritol 888 ATO	EtOH: Chloroform (20:80% *v*/*v*)		Drug, 5 mL lipids	20 mL aqueous solution 3% *w*/*v* Tween 80 or 2.5% Tween 80 and 0.5% *w*/*v* polyvinyl alcohol	Probe sonicator	15,000 rpm		DLS, FTIR, XRD, SEM, UV-Vis, in vitro drug release, in vivo studies	[192]
**Synthetic compounds**										
Docetaxel	Soy lecithin, monostearin	3 mL chloroform		10 mg drug, 40 mg lecithin, 100 mg monostearin, 40 mg vitamin E	10 mL deionized water, 150 mg tween 80	15 min	11,000 rpm 3 min	3 h with stirring	XPS, PCS, HPLC, PXRD, in vitro drug release, cytotoxicity, in vivo studies	[193]
Haloperidol	Glyceryl monostearate, Compritol ATO 888, precirol ATO 5, stearic acid or palmitic acid	2.5 mL chloroform: EtOH 1:1 *v*/*v*		43.75–50 mg Drug, 87.5–100 mg lipid	22.5 mL aqueous solution of Tween 80 (1.5–1.625% *w*/*v*)	5 min 100% amplitude	3000 rpm	30 min	PCS, TEM, UV-Vis, XRD, DSC, HPLC, in vitro drug release, in vivo studies	[182]
Levofloxacin-Doxycycline	Compritol 888 ATO 2.5–4.5%, stearic acid 1–2%			Drug, lipids, 1.75–2.5% Span 60 (emulsifier)	Distilled water, 0.5% Pluronic F127 (emulsifier)	10 cycles 1 min on/off	24,000 rpm		DLS, in vitro drug release, UV-Vis, TEM, FTIR, DSC, CLSM, ex vivonasal permeation, HPLC, in vivo studies	[183]
Lipophilic Kiteplatin Pt(IV) Prodrugs (SMF 111, 196, 200, 144)	Cetyl palmitate (lipid matrix), 16:0 PEG-2-PE (surface-modifier)	1 mL chloroform	65 °C	Drug, lipids	3 mL ultrapure water, Tween 80 3% p/V (surface modifier)	Probe-tip 0.27 W	Gently stirred	15 min	H-NMR, AAS, in vitro drug release, FLM, UV-Vis, DLS, TEM	[194]

Abbreviations: 16:0 PEG-2-PE—1,2-Dipalmitoylsn-glycero-3-phosphoethanolamine-N-[methoxy (poly (ethylene glycol)-2000]; AAS—Atomic Absorption Spectroscopy; AFM—Atomic Force Microscopy; BACE1 siRNA—Beta-site APP-cleaving enzyme 1 small interfering RNA; CLSM—Confocal Laser Scanning Microscopy; DCM—Dichloromethane; DLS—Dynamic Light Scattering; DSC—Differential Scanning Calorimetry; EtOH—Ethanol; FMPR—Fluorescence Microplate Reader; FTIR—Fourier-transform Infrared Spectroscopy; H-NMR—Proton Nuclear Magnetic Resonance; HPLC—High-Performance Liquid Chromatography; HPLC-DAD—High-Performance Liquid Chromatography coupled to Diode Array Detection; HPLC-FLD—High Performance Liquid Chromatography with Fluorescence Detection; PCS—Photon Correlation Spectroscopy; PXRD—Powder X-ray Diffraction; SEM—Scanning Electron Microscopy; SMF 111—cis,trans,cis [PtCl_2_{O_2_C(CH_2_)_4_CH_3_}_2_(cis-1,4-diaminocyclohexane)]; SMF 144—cis,trans,cis[PtCl_2_{O_2_C(CH_2_)_8_CH_3_}_2_(cis-1,4- diaminocyclohexane)]; SMF 196—cis,- trans,cis[PtCl_2_{O_2_CCH_3_)_2_CH_3_}_2_(cis-1,4-diaminocyclohexane)]; SMF 200—cis,trans,cis[PtCl_2_{O_2_C(CH_2_)_2_CH_3_}_2_(cis-1,4-diaminocyclohexane)]; T—Temperature; TEM—Transmission Electron Microscopy; UV-Vis —Ultraviolet-visible spectroscopy; XPS—X-ray Photoelectron Spectroscopy; XRD—X-ray Diffraction.

**Table 5:** NLCs produced to encapsulate neuroprotective compounds and extracts.

Compounds/ Extracts	Lipids	Solvent	T	Oil Phase	Aqueous Phase	Ultrasound	Rotation	Time	Analysis	Ref
**Seaweed extract**										
*Sargassum longifolium* (Turner) C.Agardh	Lecithin, stearic acid, orange oil	EtOH: acetone (40:60 *v*/*v*)	72–75 °C	Lipids	1% *w*/*v* Poloxamer 188		Yes		DLS, SEM, TEM, FTIR, UV-Vis, in vitro drug release, cytotoxicity	[71]
**Natural compounds**										
Astaxanthin	Glyceryl behenate as solid lipid, oleic acid as liquid lipid	PBS for Tween 80	78 °C	Lipids, drug, lecithin	Tween 80, PBS	15 min at 25 °C; 4 min 2 s on/ off	2000 rpm	3 min	PCS, UV-Vis, XRD, DSC	[185]
Baicalin, Salvianolic acid B	Lecithin, Compritol 888 ATO	EtOH for drug and lecithin, chloroform for compritol 888 ATO, mPEG-MAL, mPEG-OH and MCT 812	75 °C	Drug, lipids, mPEG-MAL, mPEG-OH, MCT 812	Myrj 52 dissolved in deionized water		Yes	2 h	PCS, HPLC, in vitro drug release, UPLC, in vivo studies	[195]
Resveratrol	Cetyl palmitate, Capmul MCM		5 °C above solid lipid melting point	Drug, lipids, Acrysol K150	Distilled water, Poloxamer 188, Tween 80	Probe 2 min 30% amplitude, 3s on, 2 off	Yes	2 min	DLS, HPLC, TEM, DSC, FTIR, in vivo studies	[196]
**Synthetic compounds**										
Almotriptan maleate	Compritol 888 ATO, Precirol ATO 5 or stearic acid as solid lipid, Labrafil M2125CS as liquid lipid		77 °C	Drug, lipids	Water, Tween 80: (Lauroglycol, Labrasol or Transcutol) 2:1 (3.5% *w*/*v*)	15 min	Yes	10 min	PCS, UV-Vis, DSC, in vitro drug release, ex vivo drug permeation, in vivo studies	[189]
Asenapine	Glyceryl monostearate (800 mg), oleic acid (160 mg)		70 °C	80 mg drug, lipids	50 mL aqueous solution 1.5% *w*/*v* Tween 80	5 min 60% amplitude, on/off 0.5 s	16,000 rpm		DLS, HPLC, DSC, XRD, FTIR, TEM, AFM, in vitro drug release, in vivo studies	[197]
Carbamazepine	Trilaurin, oleic acid	DMSO for drug	70 °C	Drug, lipids, surfactants (Tween 80, Span 80 and Poloxamer 188)	Deionized water		800 rpm	30 min	DLS, UV-Vis, TEM, DSC, PXRD, FTIR, HPLC, invitro drug release, in vivo studies	[198]
Efavirenz	Precirol ATO 5, Captex P 500 (8:2; 7:3)		66 °C	Drug, lipids	Deionized water, MYS-25 (1; 2% *w*/*v*)	30 s on, 5 off, 75 or 90% amplitude		4 min	HPLC, DLS, TEM, FTIR, DSC, PXRD, in vitro drug release, in vivo studies	[199]
Lopinavir	Compritol 888, oleic acid (60:40 to 80:20)		80 °C	Drug, lipids	Water, Tween 80	50% amplitude 5 min	1200 rpm	15 min	DLS, UV-Vis, in vitro drug release, TEM, in vivo studies	[200]
Lurasidone hydrochloride	Gelot 64, Capryol 90	EtOH: acetone 1:1		Drug, lipids	Distilled water, Tween 80, Transcutol P	Probe 6 min	Continuous	2 h	PCS, UV-Vis, TEM, SEM, DSC, HPLC, in vitro drug release, in vivo studies	[201]
Ondansetron hydrochloride	Glyceryl monostearate, Capryol 90, soy lecithin		85 °C	Drug, glyceryl monostearate, capryol 90	Poloxamer 188, soy lecithin, water		1500 rpm	10 min	DLS, UV-Vis, in vitro drug release, ex vivo permeation, SEM, DSC, XRD, in vivo studies	[184]
Rivastigmine	Glyceryl monostearate, Capmul MCM C8 3:2	1 mL EtOH	70 °C	Drug, lipids, 0.1% stearylamine, lecithin	Double distilled water, Tween 80	180 W, 2 min, 4 s pulses, 3 s off	Yes	2 h	UV-Vis, HPLC, in vivo studies	[202]

Abbreviations: AFM—Atomic Force Microscopy; DLS—Dynamic Light Scattering; DSC—Differential Scanning Calorimetry; FTIR—Fourier-transform Infrared Spectroscopy; HPLC—High-Performance Liquid Chromatography; MCT—Medium Chain Triglycerides; mPEG-OH—Methoxypolyethylene glycol; mPEG-MAL—Methoxypolyethylene glycol maleimide; PCS—Photon Correlation Spectroscopy; PXRD—Powder X-ray Diffraction; SEM—Scanning Electron Microscopy; TEM—Transmission Electron Microscopy; UPLC—Ultra Performance Liquid Chromatography; UV-Vis—Ultraviolet-visible spectroscopy; XRD—X-ray Diffraction.
